# Targeting IMPDH to inhibit SAMHD1 in *KMT2A*-rearranged leukaemia

**DOI:** 10.1080/15384101.2025.2601796

**Published:** 2025-12-15

**Authors:** Yolande Klootsema, Nikolaos Tsesmetzis, Sushma Sharma, Sophia Hofmann, Jonas Thier, Christopher Dirks, Femke M. Hormann, Miriam Yagüe-Capilla, Anna Bohlin, Sofia Bengtzen, Sören Lehmann, Andrei Chabes, Martin Jädersten, Vanessa Lundin, Sean G. Rudd, Ingrid Lilienthal, Nikolas Herold

**Affiliations:** a Division of Paediatric Oncology and Surgery, Department of Women’s and Children’s Health, Karolinska Institutet, Stockholm, Sweden; b Department of Medical Biochemistry and Biophysics, Umeå University, Umeå, Sweden; c Center for Haematology and Regenerative Medicine, Department of Medicine Huddinge, Karolinska Institutet, Huddinge, Sweden; d Science for Life Laboratory (SciLifeLab), Department of Oncology-Pathology, Karolinska Institutet, Stockholm, Sweden; e Instituto de Biomedicina de Sevilla (IBiS), Hospital Universitario Virgen del Rocío/CSIC/Universidad de Sevilla, Sevilla, Spain; f Departamento de Biología Celular, Facultad de Biología, Universidad de Sevilla, Sevilla, Spain; g Department of Medicine, Division of Hematology, Karolinska University Hospital, Huddinge, Sweden; h Department of Oncology-Pathology, Karolinska Institutet, Stockholm, Sweden; i Hematology Unit, Department of Medical Sciences, Uppsala University, Uppsala, Sweden; j Paediatric Oncology, Astrid Lindgren Children’s Hospital, Karolinska University Hospital, Stockholm, Sweden

**Keywords:** IMPDH, KMT2A, leukemia, SAMHD1, therapy resistance

## Abstract

Cytarabine (ara-C) and fludarabine (F-ara-A) are key drugs in leukaemia treatment. SAMHD1 is known to confer resistance to ara-C and F-ara-A, and we previously identified ribonucleotide reductase inhibitors as indirect SAMHD1 inhibitors in a phenotypic screen. The inosine monophosphate dehydrogenase (IMPDH) inhibitor mycophenolic acid (MPA) was also a hit in this screen. IMPDH inhibitors (IMPDHi) have previously shown efficacy against *KMT2A*-rearranged (*KMT2A*r) acute myeloid leukaemia (AML). We investigated whether IMPDH inhibition could enhance the effect of ara-C and F-ara-A in AML cell lines and primary AML samples, and whether this effect was linked to *KMT2A* status. We found that sensitivity to IMPDHi was independent of *KMT2A* status. IMPDHi synergized with ara-C and F-ara-A in a SAMHD1-dependent manner in a subset of AML cells, but not in acute lymphoblastic leukaemia cell lines. Mechanistically, IMPDHi depleted allosteric SAMHD1 activators GTP and dGTP, thereby increasing active triphosphate metabolites in SAMHD1-proficient, but not SAMHD1-deficient, cells. Our findings suggest that the addition of IMPDHi to ara-C and F-ara-A may have therapeutic benefits in some AML cases.

## Introduction

Rearrangements of the lysine methyltransferase 2A gene *KMT2A* (*KMT2A*r) are a recurrent genetic event in both acute lymphoblastic leukaemia (ALL) and acute myeloid leukaemia (AML) [1]. With up to 80% of cases, the prevalence of *KMT2A*r is particularly high in infant ALL [2]. While outcomes in *KMT2A*r infant ALL are still inferior to non-*KMT2A*r infant ALL and pediatric ALL, incorporation of cytarabine (ara-C) to induction treatment and high-dose ara-C during consolidation have resulted in 3-year event-free survival (EFS) of 66% [3]. *In vitro*, *KMT2A*r ALL is particularly sensitive to ara-C [4]. In AML, with the exception of *KMT2A:MLLT3*, *KMT2A*r belongs to the adverse risk group according to the ELN-2022 criteria and should therefore undergo allogeneic haematopoietic stem cell transplantation following intensive ara-C-based chemotherapy [5]. Hence, ara-C plays an important role in the treatment of *KMT2A*r leukaemia, but other nucleoside analogues such as fludarabine (F-ara-A), clofarabine (Cl-F-ara-A) and cladribine (2-CdA) are also increasingly used in both primary treatment and the relapsed and refractory setting [6]. We and others have previously described SAMHD1’s ara-CTPase activity as a major resistance factor against ara-C and other nucleoside analogues associated with worse outcomes in AML [7–11]. SAMHD1 is an allosterically regulated homotetramer that requires binding of (deoxy)guanosine triphosphate ((d)GTP) and a deoxynucleoside triphosphate (dNTP) to allosteric sites 1 and 2, respectively [12]. In a phenotypic screen using the *KMT2A*r AML cell line THP-1, we identified that noncompetitive irreversible inhibitors of ribonucleotide reductase (RNR), like gemcitabine, can indirectly suppress the ara-CTPase activity of SAMHD1 [13]. The proposed mechanism of action is a change in the dNTP pool balance, leading to a shift from dATP- to dCTP-activated SAMHD1, which results in loss of enzymatic activity toward ara-CTP. We recently demonstrated the safety of incorporating the RNR inhibitor hydroxyurea (HU) into front-line intensive ara-C-based chemotherapy and the ability to increase ara-CTP levels in circulating leukaemic cells in a phase 1 clinical trial [14].

Recently, Liu *et al*. reported *KMT2A*r AML to be selectively vulnerable to inhibition of inosine monophosphate dehydrogenase (IMPDH), resulting in apoptosis [15]. Interestingly, the IMPDH inhibitor mycophenolic acid (MPA) was another hit in our phenotypic screen to identify SAMHD1 inhibitors [13]. As IMPDH is the rate-limiting enzyme for *de novo* (deoxy)guanosine triphosphate ((d)GTP) synthesis, we hypothesized that MPA might reduce SAMHD1 activity through depletion of allosteric site 1 activators, as a result of IMPDH inhibition. Here, we used a panel of *KMT2A*r and non-*KMT2A*r leukaemic cell lines, haematopoietic stem and progenitor cells (HSPCs) differentiated from *KMT2A*r-induced pluripotent stem cells (iPSCs), and *KMT2A*r AML primary patient samples to investigate to what extent IMPDHi-mediated suppression of SAMHD1 activity can sensitize leukaemic cells to ara-C, F-ara-A and other nucleoside analogues and whether this is affected by the *KMT2A* status.

The main hypothesis of the current work was that depletion of the allosteric SAMHD1 activator (d)GTP would inhibit SAMHD1 activity toward antileukaemic nucleoside analogue triphosphates.

## Results

### KMT2A status does not correlate with sensitivity to IMPDH inhibitors

**Figure 1.**
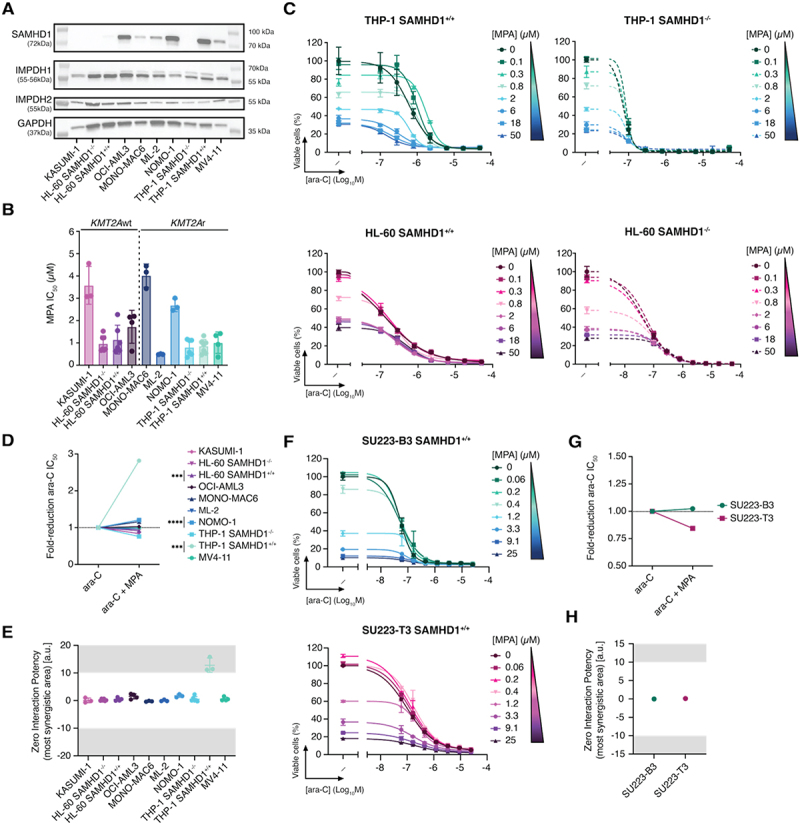
*KMT2A*-rearranged AML is not selectively sensitive to MPA but shows ara-C synergy in a subset of cell lines. (A) Protein expression of SAMHD1, IMPDH1, and IMPDH2 by western blot. (B) MPA ic_5__0_ values across AML cell lines. Bars indicate mean from a minimum of three independent experiments, with error bars showing sd and points corresponding to individual experiments. (C, F) representative dose – response curves of ara-C combined with MPA in (B) THP-1 and HL-60 cells with or without SAMHD1 or (E) iPscs cells. Data are mean ± SD from one experiment representative of a single or three biological replicates. (D, G) maximum fold-reduction in ara-C ic_5__0_ upon co-treatment with MPA in (C) AML models and (F) iPscs based on single or three independent experiments. To avoid confounding effects from cytotoxicity, only concentrations at which MPA alone reduced viability by ≤80% were included. Each dot represents the mean fold reduction in ara-C ic_5__0_ across one or three independent experiments, based on the combination condition that yielded the greatest reduction. MPA concentrations used for each model were as follows: THP-1 SAMHD1^+/+^ and THP-1 SAMHD1^−/−^, 50 µM; HL-60 SAMHD1^+/+^, HL-60 SAMHD1^−/−^, and OCI-AML3, 0.1 µM; MV4-11, 0.03 µM; NOMO-1, 0.3 µM; MONO-MAC6, 20 µM; ML-2, 0.8 µM; KASUMI-1, 0.1 µM. (E, H) ZIP synergy scores from the most synergistic area for combinations of ara-C + MPA in (D) AML models or (G) iPscs. Synergy was assessed using the ZIP model, with scores corresponding to the regions predicted to exhibit maximal synergy; corresponding analyses using Bliss, Loewe, and HSA models are presented in Supplementary Figure 7 and Supplementary Table 2. Values shown as mean ± SD with each point representing individual values from one or three biological replicates. Data information: for (D) Statistical significance was assessed using unpaired two-tailed t-tests (*p < 0.05, **p < 0.01, ***p < 0.001, ****p < 0.0001); the absence of symbols indicates that statistical analysis was not significant or not performed (see Methods). Detailed results for each maximum fold-reduction in ara-C IC_50_ are as follows: Figure 1(D) – ara-C vs ara-C + MPA: THP-1 SAMHD1^+/+^, *n* = 3, *P* = 0.003, *t* = 11.8, *df* = 4; THP-1 SAMHD1^−/−^, *n* = 3, *P* = 0.2479, *t* = 1.352, *df* = 4; HL-60 SAMHD1^+/+^, *n* = 3, *P* = 0.003, *t* = 11.76, *df* = 4; HL-60 SAMHD1^−/−^, *n* = 3, *P* = 0.1448, *t* = 1.809, *df* = 4; OCI-AML3, *n* = 3, *P* = 0.3373, *t* = 1.089, *df* = 4; MV4-11, *n* = 3, *P* = 0.9691, *t* = 0.04123, *df* = 4; NOMO-1, *n* = 3, *P* =  < 0.0001, *t* = 17.98, *df* = 4; MONO-MAC6, *n* = 3, *P* = 0.2786, *t* = 1.252, *df* = 4; ML-2, *n* = 3, *P* = 0.3998, *t* = 0.9413, *df* = 4; KASUMI-1, *n* = 3, *P* = 0.5368, *t* = 0.6748, *df* = 4. For individual ic_5__0_ values compared across multiple cell lines, a Kruskal – Wallis test was performed; although some significant differences were observed, they are not shown, as they did not provide additional information beyond fold-reduction analyses (see Methods).

*KMT2A*r AML cell lines were previously reported to be significantly more sensitive to IMPDH inhibition as compared to *KMT2A*wt cells [15]. To test this in our system, we treated a panel of *KMT2A*r and *KMT2A*wt AML cell lines that differed in their levels of SAMHD1 expression (Figure 1(A)) with various IMPDH inhibitors: the noncompetitive inhibitor MPA [16], the competitive inhibitors ribavirin (RBV) [17] and bredinin aglycone (BAg, mizoribine, FF-10501–01) [18], and the irreversible inhibitor disulfiram (DIS) [19]. The AML cell lines used had similar IMPDH1 and IMPDH2 protein levels (Figure 1(A)). Cell viability at increasing drug concentrations was determined using a luciferase-based ATP-release assay, and full dose-response curves were fitted to determine half-maximal inhibitory concentrations (IC_50_). Surprisingly and in contrast to a previous report [15], the IC_50_ values for the IMPDH inhibitors tested did not correlate with *KMT2A* status, with a range of ~0.5 to ~4 µM irrespective of *KMT2A*r (Figure 1(B); Fig S1A; Fig S2A). No correlation with SAMHD1 expression was observed either (Figure 1(B); Fig S1A; Fig S2A).

### Ara-C and F-ara-A synergize with MPA and ribavirin in a subset of AML cell lines

As our phenotypic screen for SAMHD1 inhibitors to overcome ara-C resistance, which identified RNR inhibitors [13], also yielded MPA as a candidate, and SAMHD1 is a resistance factor for ara-C, we hypothesized that IMPDH inhibition could enhance the efficacy of ara-C. Using cell viability assays, we found that some SAMHD1-expressing cells were more sensitive to ara-C in the presence of MPA, with an ara-C IC_50_ reduction of up to three-fold in SAMHD1-expressing THP-1 cells but not in their SAMHD1-deficient counterparts (Figure 1(C,D)). To assess drug synergy, we calculated Zero Interaction Potency (ZIP) scores [20] using Synergy Finder software [21], with ZIP scores > 10 or > 5, depending on the definition, indicating drug synergism [22]. In addition, we used the Bliss independence, Loewe additivity, and Highest Single Agent (HSA) models, which yielded similar results in all experiments (see Fig S7 and Table S2). This analysis reflected synergy between ara-C and MPA in SAMHD1-proficient THP-1 cells, with a mean ZIP score of 12.8 (Figure 1(E)). Synergy was not observed in SAMHD1-deficient THP-1 cells (Figure 1(E)). No synergistic effect on ara-C efficacy was observed in the presence of MPA in the additional eight cell lines tested, instead these drugs combined in an additive manner (Figure 1(C-E); Fig S1B). In summary, MPA synergized with ara-C in a SAMHD1-dependent manner in THP-1 cells but did not alter the ara-C efficacy in the other cell lines tested, regardless of SAMHD1- or *KMT2A*-status.

To further study the impact of *KMT2A*r on MPA/ara-C interactions, we differentiated patient-derived iPSCs [23] with wild-type (SU223-T3) or rearranged (SU223-B3) *KMT2A* into AML-like cells. While the *KMT2A*r cells had a 1.2-fold lower IC_50_ of ara-C than their *KMT2A*wt counterparts, they had a similar IC_50_ of MPA (1.2 μM for SU223-T3 and 0.83 μM for SU223-B3) (Figure 1(F)). We observed no synergism between MPA and ara-C in these cells (Figure 1(F-H)). These results suggest that *KMT2A*-rearrangement does not cause ara-C/MPA synergy in iPSC-derived AML cells.

**Figure 2.**
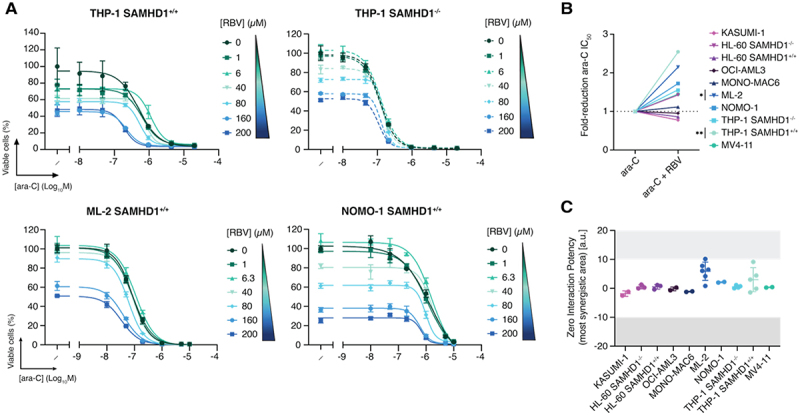
Ara-C and RBV synergize in AML cell lines. (A) Representative dose – response curves of ara-C combined with RBV in THP-1 SAMHD1^+/+^, THP-1 SAMHD1^−/−^, ML-2 and NOMO-1 cells. Points and error bars indicate mean ± SD from one experiment representative of three biological replicates.(B) Maximum fold-reduction in ara-C ic_5__0_ upon co-treatment with RBV, based on three independent experiments. To avoid confounding effects from cytotoxicity, only concentrations at which RBV alone reduced viability by ≤80% were included. Each dot represents the mean fold reduction in ara-C ic_5__0_ across three independent experiments, based on the combination condition that yielded the greatest reduction. RBV concentrations used for each model were as follows: THP-1 SAMHD1^+/+^, THP-1 SAMHD1^−/−^, MONO-MAC6, and ML-2: 200 µM; NOMO-1: 160 µM; HL-60 SAMHD1^+/+^, HL-60 SAMHD1^−/−^, OCI-AML3, MV4-11, and KASUMI-1: 1 µM.(C) ZIP synergy scores from the most synergistic area for ara-C + RBV, shown as mean ± SD with each point representing individual values from at least two independent experiments. Synergy was evaluated using the ZIP model, with reported scores reflecting the regions of maximal predicted synergy. Results from Bliss, Loewe, and HSA models are included in supplementary Figure 7 and Supplementary Table 2.

The competitive IMPDHi RBV sensitized THP-1 cells to ara-C in a SAMHD1-dependent manner, with a two-fold reduction in the IC_50_ of ara-C (Figure 2(A,B)). Similar results were obtained in the SAMHD1-positive, *KMT2Ar* AML cell line ML-2 (Figure 2(A,B)). Synergism, albeit with scores below 10, was also evidenced by ZIP scores in THP-1 SAMHD1^+/+^ and ML-2 (Figure 2(C)). No ara-C sensitization or RBV/ara-C synergy was observed for additional cell lines tested, which instead demonstrated an additive response (Figure 2(A,B); Fig S1C).

Data information: For (B) Statistical significance was assessed using unpaired two-tailed t-tests (*p < 0.05, **p < 0.01, ***p < 0.001, ****p < 0.0001); the absence of symbols indicates that statistical analysis was not significant or not performed (see Methods). Detailed results for each maximum fold-reduction in ara-C IC_50_ are as follows: Figure 2(B) – ara-C vs ara-C + RBV: THP-1 SAMHD1^+/+^, *n* = 3, *P* = 0.0042, *t* = 5.887, *df* = 4; THP-1 SAMHD1^−/−^, *n* = 3, *P* = 0.0607, *t* = 2.59, *df* = 4; HL-60 SAMHD1^+/+^, *n* = 3, *P* = 0.5179, *t* = 0.7089, *df* = 4; HL-60 SAMHD1^−/−^, *n* = 3, *P* = 0.2974, *t* = 1.197, *df* = 4; ML-2, *n* = 3, *P* = 0.0150, *t* = 4.092, *df* = 4.

To assess whether other inhibitors of IMPDH could synergize with ara-C, we combined BAg or DIS with ara-C. Neither BAg nor DIS increased ara-C efficacy across the AML cell lines tested and instead combined in an additive manner (Fig S2B-G).

**Figure 3.**
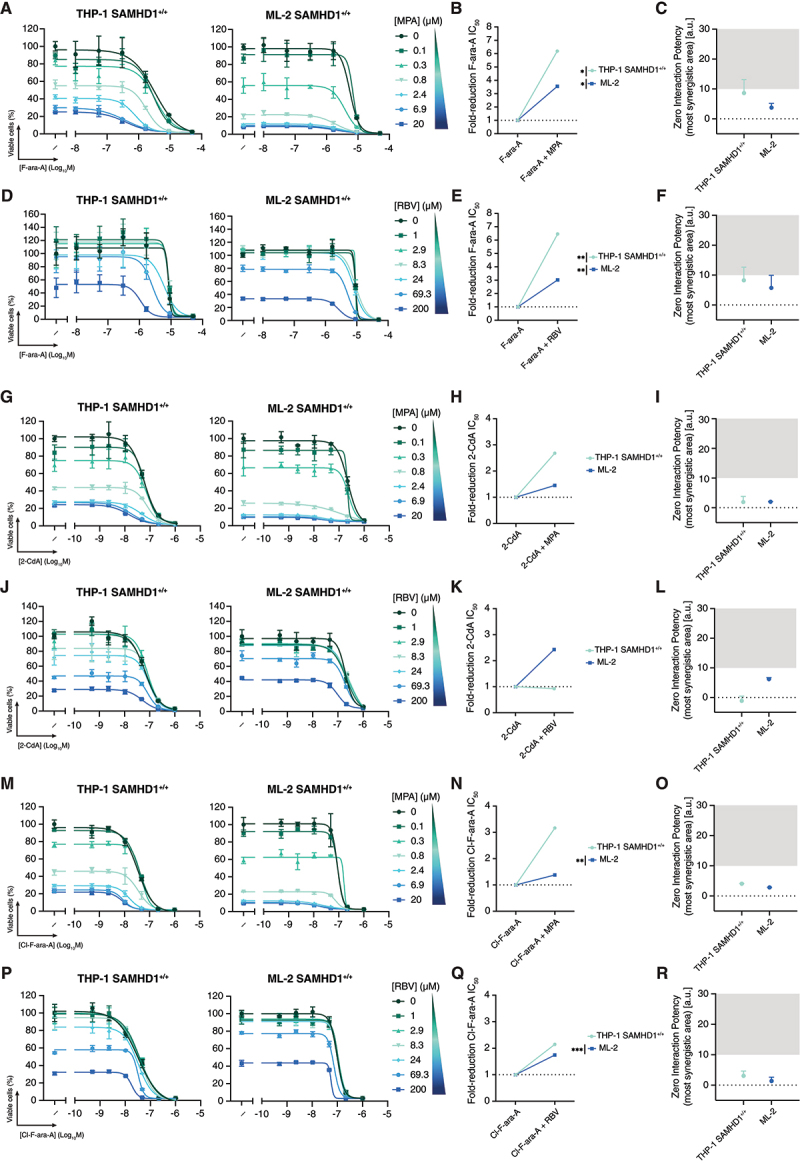
IMPDH inhibition differentially modulates fludarabine, cladribine and clofarabine sensitivity (A, D, G, J, M, P) Representative dose – response curves of fludarabine (F-ara-A), cladribine (2-CdA), and clofarabine (Cl-F-ara-A) combined with either MPA (panels A, G, M) or RBV (panels D, J, P) in THP-1 SAMHD1^+/+^ and ML-2. Data are mean ± SD. (B, E, H, K, N, Q) maximum fold-reduction in F-ara-A, 2-CdA, and Cl-F-ara-A ic_5__0_ upon co-treatment with MPA or RBV. To avoid confounding effects from cytotoxicity, only concentrations at which MPA or RBV alone reduced viability by ≤80% were included. Each point represents the mean fold reduction in F-ara-A, 2-CdA, or Cl-F-ara-A ic_5__0_ across several independent experiments, based on the combination condition that yielded the greatest reduction. Concentrations of MPA or RBV used in THP-1 SAMHD1^+/+^ and ML-2 were as follows: F-ara-A combined with MPA – THP-1 SAMHD1^+/+^ and ML-2, 20 µM; F-ara-A combined with RBV – THP-1 SAMHD1^+/+^ and ML-2, 200 µM; 2-CdA combined with MPA – THP-1 SAMHD1^+/+^ , 20 µM; and ML-2, 0,83 µM; 2-CdA combined with RBV – THP-1 SAMHD1^+/+^ and ML-2, 200 µM; Cl-F-ara-A combined with MPA – THP-1 SAMHD1^+/+^ , 20 µM; and ML-2, 0,83 µM; Cl-F-ara-A combined with RBV – THP-1 SAMHD1^+/+^ and ML-2, 200 µM. (C, F, I, l, O, R) ZIP synergy scores from the most synergistic area for combinations of F-ara-A, 2-CdA or Cl-F-ara-A combined with either (C, I, O) MPA or (F, L, R) RBV. Synergy was evaluated using the ZIP model, with reported scores reflecting the regions of maximal predicted synergy. Results from Bliss, Loewe, and HSA models are included in Supplementary Figure 7 and Supplementary Table 2. Values shown as mean ± SD with each point representing individual values from several biological replicates.

To examine whether IMPDH inhibition could potentiate additional nucleoside analogues beyond ara-C, we evaluated combinations of MPA or RBV with F-ara-A, 2-CdA, and Cl-F-ara-A in THP-1 SAMHD1^+/+^ and ML-2 cells, the two lines previously showing synergy with ara-C. The strongest effect was observed for F-ara-A, with MPA reducing its IC_5__0_ by 3.5-fold in ML-2 and up to 6-fold in THP-1 SAMHD1^+^/^+^ (Figure 3(A,B)), with ZIP scores > 5 indicating synergy (Figure 3(C)). RBV produced similar potentiation (Figure 3(D)), lowering F-ara-A IC_5__0_ by ~6.5-fold and 3-fold in THP-1 SAMHD1^+^/^+^ and ML-2, respectively (Figure 3(E)), consistent with ZIP analysis (Figure 3(F)).

Data information: For B,E,H,K,N,Q, statistical significance was assessed using unpaired two-tailed t-tests (*p < 0.05, **p < 0.01, ***p < 0.001, ****p < 0.0001); the absence of symbols indicates that statistical analysis was not significant or not performed (see Methods). Detailed results for each maximum fold-reduction in F-ara-A IC_50_ are as follows: Figure 2(B) – F-ara-A vs F-ara-A + MPA: THP-1 SAMHD1^+/+^, *n* = 3, *P* = 0.0122, *t* = 4.348, *df* = 4. F-ara-A vs F-ara-A + MPA: ML-2, *n* = 3, *P* = 0.0139, *t* = 4.182, *df* = 4. Figure 2E – F-ara-A vs F-ara-A + RBV: THP-1 SAMHD1^+/+^, *n* = 3, *P* = 0.0093, *t* = 4.703, *df* = 4. F-ara-A vs F-ara-A + RBV: ML-2, *n* = 3, *P* = 0.0030, *t* = 6.433, *df* = 4. Figure 2H–2-CdA vs 2-CdA + MPA: THP-1 SAMHD1^+/+^, *n* = 2, *P* = 0.3140, *t* = 1.333, *df* = 2. 2-CdA vs 2-CdA + MPA: ML-2, *n* = 2, *P* = 0.2982, *t* = 1.393, *df* = 2. Figure 2K–2-CdA vs 2-CdA + RBV: THP-1 SAMHD1^+/+^, *n* = 2, *P* = 0.8434, *t* = 0.2242, *df* = 2. 2-CdA vs 2-CdA + RBV: ML-2, *n* = 2, *P* = 0.0567, *t* = 4.021, *df* = 2. Figure 2N – Cl-F-ara-A vs Cl-F-ara-A + MPA: THP-1 SAMHD1^+/+^, *n* = 2, *P* = 0.1277, *t* = 2.523, *df* = 2. Cl-F-ara-A vs Cl-F-ara-A + MPA: ML-2, *n* = 2, *P* = 0.0080, *t* = 11.11, *df* = 2. V 2Q – Cl-F-ara-A vs Cl-F-ara-A + RBV: THP-1 SAMHD1^+/+^, *n* = 2, *P* = 0.0983, *t* = 2.949, *df* = 2. Cl-F-ara-A vs Cl-F-ara-A + RBV: ML-2, *n* = 2, *P* = 0.0004, *t* = 53.30, *df* = 2.

In contrast, co-treatment of 2-CdA with MPA only modestly increased sensitivity in THP-1 SAMHD1^+/+^ and ML-2 cells. When paired with RBV, the effect was largely additive (Figure 3(J)), with the greatest IC_5__0_ reduction for 2-CdA observed in ML-2 cells (Figure 3(K)), consistent with ZIP scores < 5 (Figure 3(L)). Similarly, Cl-F-ara-A showed limited sensitization (Figure 3(M)): in THP-1 SAMHD1^+/+^ cells, MPA reduced its IC_5__0_ by more than three-fold (Figure 3(N)), although synergy scores indicated an additive effect (Figure 3(O)). RBV co-treatment produced comparable additive effects in both cell lines (Figure 3(P-R)). These results are consistent with previous reports in which SAMHD1 depletion or inhibition had much weaker effects on 2-CdA and Cl-F-ara-A efficacy as compared to ara-C and F-ara-A [7,8,13].

Given a discrepancy of previously reported dependency on *KMT2A*r for IMPDHi sensitivity [15] with our data, we assessed whether previously reported synergistic effects between MPA and the Bcl-2 inhibitor venetoclax (VEN) in *KMT2A*r cells could be reproduced [15]. To this end, we tested this combination in selected AML models considering both SAMHD1 and *KMT2A* status. We found that MPA strongly reduced the VEN IC_5__0_ values regardless of *KMT2A* or SAMHD1 status, with ZIP scores indicating strong synergy in THP-1 cells (Fig S2H-J).

**Figure 4.**
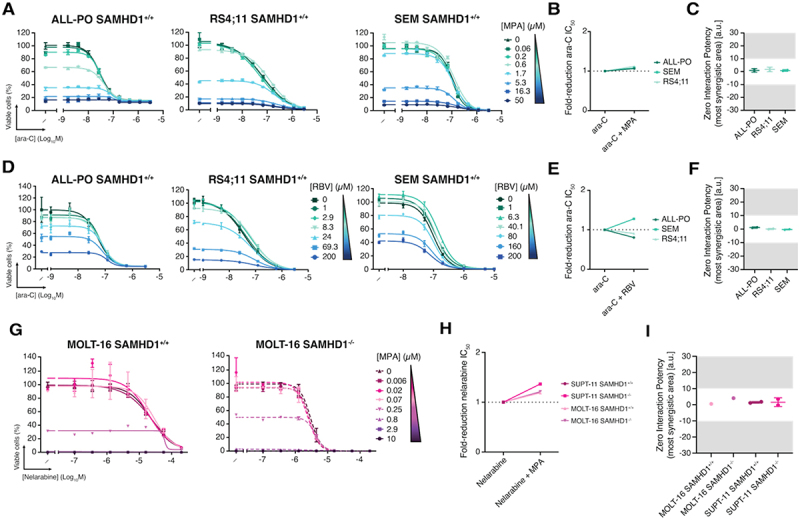
IMPDH inhibitors combined with ara-C in *KMT2A*r B-ALL and nelarabine in T-ALL. (A, D, G) dose – response curves of ara-C combined with (A) MPA, (D) RBV in B-ALL (ALL-PO, RS4;11 and SEM) and (G) nelarabine combined with MPA in T-ALL (MOLT-16 SAMHD1^+/+^ and SAMHD1^−/−^) cell lines. Each point represents the mean ± SD from one experiment representative of one or two biological replicates. (B, E, H) maximum fold-reduction in ara-C ic_5__0_ upon co-treatment with (B) MPA, (E) RBV, or (H) nelarabine ic_5__0_ combined with MPA, based on a single or two independent experiments. To avoid confounding effects from cytotoxicity, only concentrations at which MPA or RBV alone reduced viability by ≤80% were included. Each dot represents the ic_5__0_ of ara-C or nelarabine alone, or the ic_5__0_ from the combination of ara-C with MPA or RBV or nelarabine with MPA, corresponding to the condition that showed the greatest fold reduction for a single or two biological replicates. Concentrations of MPA or RBV used in each all model were as follows: ara-C combined with MPA – ALL-PO and SEM, 0.06 µM; RS4;11, 0.2 µM; ara-C combined with RBV – ALL-PO and RS4;11, 1 µM; SEM, 200 µM; nelarabine combined with MPA – MOLT16 SAMHD1^+/+^ and MOLT16 SAMHD1^−/−^, 0.02 µM. (C, F, I) ZIP synergy scores for the most synergistic area for ara-C with (C) MPA or (F) RBV and (I) nelarabine with MPA shown as mean ± SD, with each dot representing individual values from one or two independent experiments. ZIP scores, representing the regions of greatest predicted synergy, were used to assess combination effects. Corresponding analyses using Bliss, Loewe, and HSA models are presented in Supplementary Figure 7 and Supplementary Table 2.

Since *KMT2A* rearrangements are not restricted to AML, we next extended our analysis to *KMT2A*r B-cell ALL cell lines ALL-PO, RS4;11 and SEM. Across all models, neither MPA, RBV, nor BAg altered the response to ara-C and instead combined in an additive manner (Figure 4(A-F); Fig S3A-C).

Data information: For (B, C, E, F, H, I), statistical significance was assessed using unpaired two-tailed t-tests (*p < 0.05, **p < 0.01, ***p < 0.001, ****p < 0.0001); the absence of symbols indicates that statistical analysis was not significant or not performed (see Methods).

The guanosine analogue nelarabine is a common agent used to treat T-cell ALL. We hypothesized that IMPDH inhibition might be particularly effective in combination with nelarabine, as (d)GTP depletion would not only lead to SAMHD1 inhibition but also reduce competition with the active metabolite ara-GTP. However, in the T-ALL cell lines MOLT-16 and SUPT-11, MPA did not synergize with nelarabine (Figure 4(G-I); Fig S3D). We also tested nelarabine in combination with MPA or RBV in THP-1 SAMHD1^+^/^+^ and ML-2 cells. While these AML lines displayed minimal sensitivity to nelarabine, no apparent synergism was observed in the presence of MPA or RBV either (Fig. S4A, B).

### IMPDH inhibition potentiates ara-C in primary patient samples

**Figure 5.**
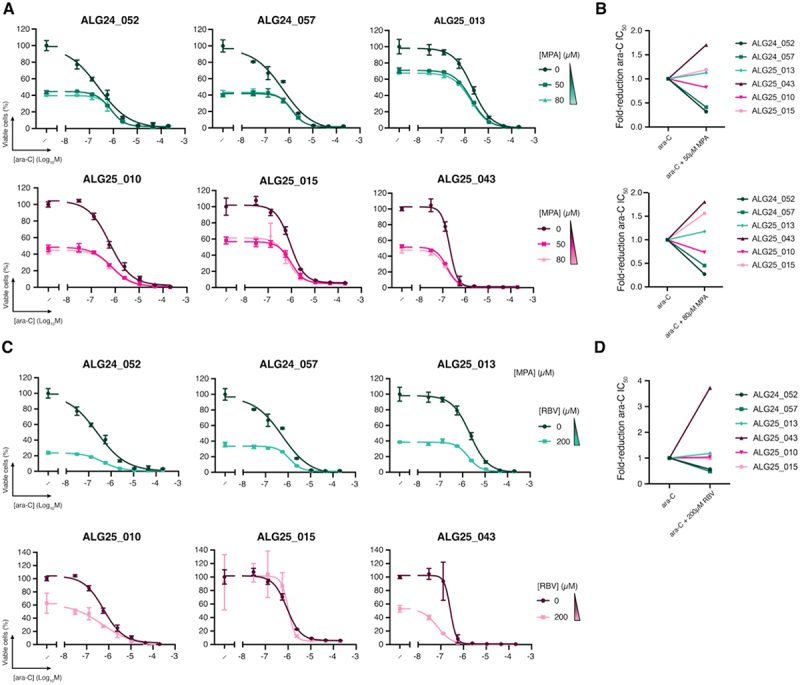
Ara-C dose-response and ic_5__0_ modulation by MPA or RBV in primary patient leukaemia samples (A, C) Representative dose – response curves of ara-C combined with MPA in primary patient leukemia cells. Patient samples ALG24_052, ALG24_057, and ALG25_013 are *KMT2A*r while ALG25_010, ALG25_015, and ALG25_043 are *KMT2A*-WT. Data are mean ± SD of three technical replicates.(B, D) maximum fold-reduction in ara-C ic_5__0_ upon co-treatment with MPA, based on one experiment. To avoid confounding effects from cytotoxicity, only concentrations at which MPA or RBV alone reduced viability by ≤80% were included. Each dot represents the fold reduction in ara-C ic_5__0_ for the combination condition tested. The concentrations used for all patient samples were: MPA, 50 µM and 80 µM; RBV, 200 µM.Patient characteristics can be found in Supplementary Table 3. ZIP scores, representing the regions of greatest predicted synergy, using Zero Interaction potency, Bliss, Loewe, and HSA models are presented in Supplementary Figure 7 and Supplementary Table 2.

Next, we sought to investigate whether IMPDH inhibition could enhance ara-C efficacy in primary AML samples from adult patients. MPA and RBV reduced the IC_50_ of ara-C by up to 1.7- and 4-fold, respectively (Figure 5(A-D)). This effect was independent of *KMT2A* status and occurred only in a subset of patients, indicating that IMPDH inhibition can sensitize primary AML cells to ara-C in select cases.

### Addition of MPA to ara-C significantly increases ara-CTP levels and depletes (d)GTP pools

**Figure 6.**
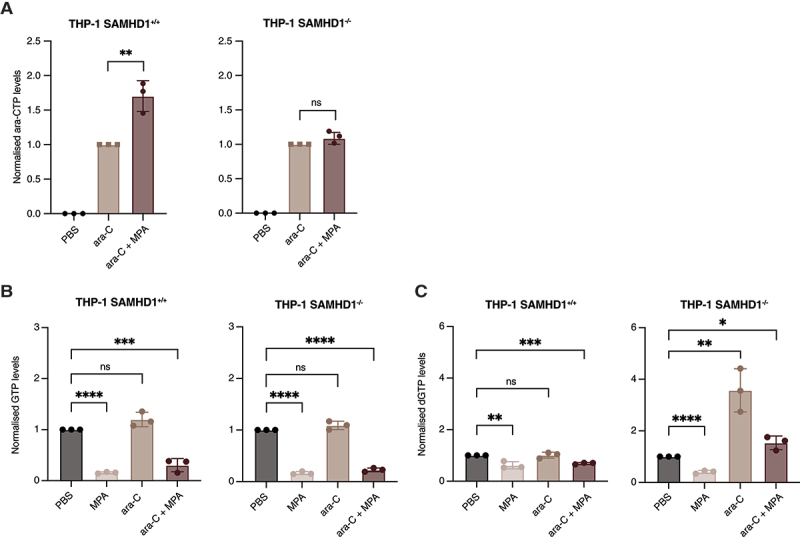
MPA-induced nucleotide pool remodeling in THP-1 cells. (A) Normalized levels of ara-CTP in THP1 SAMHD1^+/+^ and SAMHD1^−/−^ cells treated with ara-C (500 nM) with or without MPA (20 µM), normalized to CTP and then to ara-CTP levels in cells treated with ara-C alone. Data are presented as mean ± sd from three independent experiments, with each point representing an individual experiment. (B, C) normalized (B) GTP and (C) dGTP levels in SAMHD1^+/+^ and SAMHD1^−/−^ THP-1 cells following treatment with MPA (20 µM), ara-C (550 nM) or the combination of MPA (20 µM) and ara-C (500 nM), normalized to CTP and the corresponding levels in PBS-treated cells. Data are presented as mean ± SD from three independent experiments, with each point representing an individual experiment.

Ara-C undergoes triphosphorylation into its active metabolite, ara-CTP, which exerts the anti-leukaemic properties of the drug by being incorporated into nascent DNA [24]. SAMHD1 hydrolyses and inactivates ara-CTP. We therefore hypothesized that IMPDHi-mediated SAMHD1-dependent sensitization to ara-C was driven by depletion of the allosteric site 1 (AS1) activators, GTP and dGTP, resulting in reduced hydrolysis of ara-CTP. To test this, we treated SAMHD1-proficient and -deficient THP-1 cells with a sub-lethal concentration of ara-C in the absence and presence of MPA. Following 24 hours of treatment, the addition of MPA significantly increased the ara-CTP levels in cells with wild-type SAMHD1 by 1.7-fold as compared to ara-C alone (Figure 6(A)). This effect was not observed in SAMHD1-deficient cells (Figure 6(A)). As expected, we found that MPA significantly reduced GTP and dGTP levels both alone and in combination with ara-C, irrespective of SAMHD1 status (Figure 6(B,C)), consistent with its role as an IMPDH inhibitor. Interestingly, MPA treatment concomitantly led to an expansion of dTTP and dCTP pools, probably through compensatory activation of dNTP salvage pathways as described before [13] (Fig S5A, B).

Data information: For (A – C) statistical significance was assessed using unpaired two-tailed t-tests (*p < 0.05, **p < 0.01, ***p < 0.001, ****p < 0.0001); the absence of symbols indicates that statistical analysis was not significant or not performed (see Methods). Detailed results for active metabolites, GTP, dGTP pools and treatment in THP-1 wild-type (SAMHD1^+/+^) and knockout (SAMHD1^−/−^) cells are as follows: Figure 3(A) – ara-CTP (ara-C vs ara-C + MPA): THP-1 SAMHD1^+/+^, *n* = 3, *P* = 0.0055, *t* = 5.459, *df* = 4; THP-1 SAMHD1^−/−^, *n* = 3, *P* = 0.1518, *t* = 1.768, *df* = 4. Fig 3(B) - GTP (PBS vs MPA): THP-1 SAMHD1^+/+^, *n* = 3, *P* =  < 0.0001, *t* = 96.33, *df* = 4; THP-1 SAMHD1^−/−^, *n* = 3, *P* =  < 0.0001, *t* = 49.57, *df* = 4. GTP (PBS vs ara-C): THP-1 SAMHD1^+/+^, *n* = 3, *P* = 0.0744, *t* = 2.399, *df* = 4; THP-1 SAMHD1^−/−^, *n* = 3, *P* = 0.1299, *t* = 1.902, *df* = 4. GTP (PBS vs ara-C + MPA): THP-1 SAMHD1^+/+^, *n* = 3, *P* = 0.0007, *t* = 9.394, *df* = 4; THP-1 SAMHD1^−/−^, *n* = 3, *P* =  < 0.0001, *t* = 37.70, *df* = 4. Figure 3(C) - dGTP (PBS vs MPA): THP-1 SAMHD1^+/+^, *n* = 3, *P* =  < 0.0091, *t* = 4.729, *df* = 4; THP-1 SAMHD1^−/−^, *n* = 3, *P* =  < 0.0001, *t* = 17.38, *df* = 4. dGTP (PBS vs ara-C): THP-1 SAMHD1^+/+^, *n* = 3, *P* = 0.8657, *t* = 0.1803, *df* = 4; THP-1 SAMHD1^−/−^, *n* = 3, *P* = 0.0060, *t* = 5.325, *df* = 4. dGTP (PBS vs ara-C + MPA): THP-1 SAMHD1^+/+^, *n* = 3, *P* = 0.0001, *t* = 15.43, *df* = 4; THP-1 SAMHD1^−/−^, *n* = 3, *P* = 0.0243, *t* = 3.526, *df* = 4.

As no assays are available to measure SAMHD1 activity *in situ*, we resorted to a variant of our previously described *in vitro* assay [7,25] to test whether GTP and dGTP depletion would reduce SAMHD1 ara-CTPase activity. Since dGTP is a substrate for SAMHD1, we used a dilution-jump strategy to specifically monitor ara-CTP hydrolysis (Fig S6A). As expected, hydrolysis only occurred when SAMHD1 was preincubated with the AS1 and allosteric site 2 (AS2) activator dGTP, but not with the AS1-only activator GTP. However, addition of the AS2 activator dATP to GTP restored ara-CTP hydrolysis (Fig S6B). These results are in line with the notion that depletion of dGTP and GTP *in situ* would reduce SAMHD1 activity toward ara-CTP, consistent with its allosteric regulation described in prior mechanistic reports [12,26].

### Discussion

**Figure 7.**
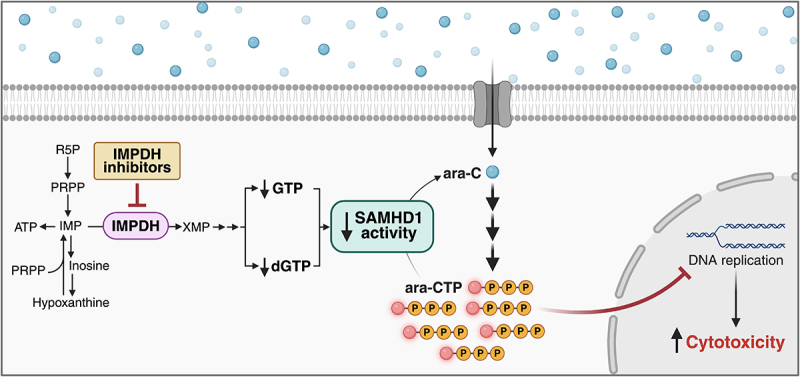
Schematic of the proposed mechanism by which IMPDH influences ara-C cytotoxicity through nucleotide pools and SAMHD1 activity. IMPDH controls guanosine nucleotide availability for SAMHD1 activation. Inhibition of IMPDH depletes GTP and dGTP, reducing SAMHD1 ara-CTPase activity, resulting in increased ara-CTP levels and enhanced cytotoxicity, as illustrated in the schematic.

In this study, we investigated whether IMPDH inhibition could sensitize leukaemic cells to ara-C and other nucleoside analogues via SAMHD1 inhibition, and whether this sensitization was dependent on *KMT2A* rearrangements. While *KMT2A*r cell lines did not exhibit increased sensitivity to IMPDH inhibition alone or in combination with ara-C or other nucleoside analogues, we could show that IMPDH inhibitors synergized with ara-C and F-ara-A in a SAMHD1-dependent manner in a subset of cell lines. This work supports a model of IMPDHi-mediated SAMHD1-dependent sensitization to ara-C and F-ara-A that is driven by depletion of SAMHD1 activators GTP and dGTP, resulting in a reduction in SAMHD1-mediated ara-C hydrolysis (Figure 7). Although our results do not support the use of IMPDH inhibition specifically for *KMT2A*r leukaemia, our findings warrant further exploration of ara-C and IMPDHi combinations, particularly with MPA.

Consistent with the identification of MPA as an IMPDHi in our phenotypic SAMHD1 inhibitor screen performed in THP-1 cells, MPA caused depletion of the allosteric site 1 activators GTP and dGTP, accompanied by expansion of ara-CTP pools. Consistent with this, *in vitro*, SAMHD1 ara-CTPase activity *in vitro* was dependent on allosteric activation by guanine nucleotides. Interestingly, inhibition of *de novo* guanosine nucleotide synthesis also resulted in increased levels of the pyrimidine deoxynucleoside triphosphates dTTP and dCTP. This is reminiscent of the effects observed with RNR inhibition, which triggers compensatory activation of the salvage pathway through dCK phosphorylation [13]. In our study, (d)GTP depletion correlated with synergistic interactions between ara-C and MPA. Similar results were observed with the IMPDHi RBV in ML-2 cells and primary AML cells. Furthermore, in addition to ara-C, F-ara-A could be potentiated by IMPDH inhibition. However, in most cell lines, only weak to moderate additive effects were observed, irrespective of *KMT2A* status or myeloid versus lymphoid origin.

IMPDH inhibition alone, therefore, does not seem to be sufficient to suppress SAMHD1 activity robustly across AML models. This may be explained by differences in baseline levels of guanosine nucleotides, which are known to differ substantially across different cell lines and haematopoietic cell types [27,28]. Alternatively, differences in activity of guanosine transport and salvage pathways for guanosine nucleotide homeostasis [29] could confer differential vulnerability for inhibition of *de novo* synthesis by IMPDHi. Hence, the net effect size of IMPDH inhibition on guanosine nucleotide levels might vary across cell types, potentially contributing to differential inhibition of SAMHD1 activity. Nevertheless, IC_50_ values for MPA and RBV were comparable between cell lines exhibiting synergy and those that did not, suggesting that baseline drug sensitivity alone does not account for the observed differences in synergistic response.

Among the four evaluated IMPDH inhibitors, only MPA and RBV enhanced the efficacy of ara-C and F-ara-A and showed synergistic activity with ara-C and F-ara-A in THP-1 and ML-2 cells, with MPA exhibiting greater potency, particularly in THP-1 cells. Of the five nucleoside analogues tested, only ara-C and F-ara-A were potentiated by IMPDH inhibition, whereas nelarabine, 2-CdA, and Cl-F-ara-A showed little or no sensitization.

The stronger effect of MPA may be attributed to its role as a noncompetitive inhibitor [16], which alters the configuration of the enzyme and cannot be overcome by increasing substrate levels. In contrast, RBV is a competitive IMPDH inhibitor [17], and its activity may be mitigated by compensatory elevation of inosine monophosphate (IMP) levels. RBV was effective in two cell lines (THP-1 and ML-2), in contrast to MPA, which showed activity only in one cell line (THP-1). This may reflect RBV’s broader mechanism of action, including perturbation of RNA capping and induction of replication stress [30], which might explain its ability to enhance ara-C cytotoxicity in contexts where MPA is ineffective. Depletion of guanosine nucleotides has also been linked to reduced glycosylation of surface proteins and decreased nitric oxide production, both of which have been associated with the immunosuppressive effects of MPA [31]. These results illustrate that compound-specific properties, beyond shared target inhibition, may critically influence drug interactions.

Myeloid and lymphoid leukaemias exhibit distinct sensitivities to antileukaemic drugs and are thus treated using lineage-specific regimens. *KMT2A*r leukaemia, on the other hand, can share properties and surface markers of both lineages, potentially requiring a combination of lymphoid- and myeloid-directed treatment [32]. Furthermore, T-ALL is particularly sensitive to the guanosine analogue nelarabine [33]. Therefore, we explored drug-drug interactions between IMDPHi and either ara-C or nelarabine in both B-ALL and T-ALL models. However, across all tested cell lines, drug interactions were largely additive, indicating that guanosine depletion alone is insufficient to potentiate nucleoside analogue cytotoxicity in these lymphoid lineages. This is in stark contrast to previous reports on SAMHD1 ara-CTPase inhibition using inhibitors of ribonucleotide reductase, observed across diverse cell lines and primary leukaemic cells [13,14].

Importantly, neither SAMHD1 expression nor *KMT2A*r status alone predicted responsiveness, emphasizing the need to integrate genetic, epigenetic, and lineage-specific features to identify predictive biomarkers and understand the mechanism of IMPDHi/ara-C synergy. Recently, Casado *et al*. used phosphoproteomic profiling to distinguish two subgroups of *KMT2A*r leukaemia, MLLGA and MLLGB. Interestingly, MLLGA was significantly more sensitive to the IMPDHi AVV-944, the RNRi gemcitabine, and ara-C [34], highlighting the importance of cellular context in shaping drug responses. Future studies combining metabolic profiling, nucleotide flux measurements, replication stress assessment, and mapping of apoptotic dependencies will be essential to define the precise contexts in which IMPDH inhibition most effectively potentiates ara-C activity. Together, these insights may inform the development of more tailored and effective treatment strategies for high-risk and lineage-ambiguous leukaemias.

This study has several limitations. As discussed above, IMPDH inhibition was only able to potentiate ara-C and F-ara-A in a subset of patients. Hence, it is currently not possible to predict which patients might benefit most from combinations of MPA or RBV with ara-C or F-ara-A. On the other hand, our data does not suggest that the efficacy of ara-C and F-ara-A might be compromised by addition of an IMPDH inhibitor as additive effects were observed even in the absence of synergy. Furthermore, we could not confirm that sensitivity of IMPDH inhibition was correlated with *KMT2A* status. While routine authentication rules out cell line mix-ups, discrepancies with previous reports might be explained by different clones of the cell lines used here and previously.

In sum, this study demonstrates that inhibition of IMPDH resulting in depletion of guanosine triphosphates can contribute to allosteric inhibition of SAMHD1 activity toward antileukaemic nucleoside analogue triphosphates like ara-CTP. While this phenotype was not evident across the whole panel of tested cell lines and primary AML samples, these results suggest that modulation of nucleotide metabolism (as demonstrated previously for inhibitors of RNR [13]) can be a powerful tool to increase the potency of antimetabolic drugs in the treatment of malignancies.

## Methods

### Cell lines

All cell lines, except KASUMI-1, were obtained from ATCC and cultured in Iscove’s Modified Dulbecco’s Medium (IMDM; cat. No. 12–440-053, Thermo Fisher Scientific). KASUMI-1 cells were a kind gift from Prof. Pär Nordlund (Department of Oncology-Pathology, Karolinska Institutet) and maintained in Roswell Park Memorial Institute medium (RPMI 1640; cat. No. 21–875-034, Thermo Fisher Scientific). Culture media were supplemented with 10% or 20% heat-inactivated fetal bovine serum (cat. A5256801, Thermo Fisher Scientific), along with 10,000 units/mL penicillin and 10,000 µg/mL streptomycin (cat. No. 15,140,148, Thermo Fisher Scientific). Cells were maintained at a density of 2–5 × 10^5^ cells/mL in a humidified incubator at 37°C with 5% CO_2_.

Generation of THP-1 and HL-60 *SAMHD1* knockout lines was previously described [6]. Cell line authentication for SAMHD1-proficient and -deficient THP-1 and HL-60 lines was performed by Eurofins Genomics Europe Applied Genomics GmbH (Ebersberg, Germany). Generation of MOLT16 *SAMHD1* knockout cells was performed as described for THP-1 cells, while the SUP-T11 *SAMHD1* knockout it was generated by a core facility. The oligonucleotides encoding for SAMHD1 gRNAs for the SUP-T11 cells, were guide 1: AAACGAGACUCAUCAAGACA – AGG and guide 2: UCCAUAGAAAAUGAAAUCAC – AGG. Cell line characteristics are summarized in Supplementary Table S1.

### iPscs

The generation of patient-derived AML iPSCs has been previously described [23]. iPSCs were cultured on Matrigel hESC-Qualified Matrix (cat. No. 354,277, Corning) in mTeSR Plus (cat. No. 100–0276, StemCell Technologies) supplemented with 1% Penicillin-Streptomycin (P/S; HyClone) and passaged as clumps using EZ-LiFT Stem Cell Passaging Reagent (cat. No. SCM139, Sigma-Aldrich). Control- and AML-HSPCs were generated using the STEMdiff Hematopoietic Kit (cat. No. 05310, StemCell Technologies), with floating cells harvested on day 10 (control) or day 12 (AML), to ensure comparable stages of hematopoietic commitment. HSPCs were expanded and drug-treated in StemSpan SFEM II (cat. No. 09655, StemCell Technologies), supplemented with P/S (1%), SCF (50 ng/mL), IL-3 (10 ng/mL), TPO (50 ng/mL), IL-6 (50 ng/mL) and FLT3L (50 ng/mL); all cytokines were obtained from Thermo Fisher Scientific.

## Primary AML blasts

Experiments were approved by the regional ethical review board in Stockholm (no. 2018/38–22, no. 2017/2085–31/2, and no. 2018/464–31/2) and adhered to the WMA Declaration of Helsinki and the Department of Health and Human Services Belmont Report. Patient sample cytogenetics in Supplementary Table S3. Cryopreserved patient-derived leukemia blasts were thawed in a 37°C water bath, transferred into pre-warmed complete medium, mixed gently, counted, pelleted by centrifugation, and resuspended in the appropriate volume of cytokine-supplemented medium. Blasts were then cultured at 1 × 10^6^ cells/ml in StemPro-34 SFM supplemented with the StemPro Nutrient Supplement (10639011; Thermo Fisher Scientific) and the same additives used for cell line cultures. Media were further supplemented with sterile-filtered recombinant human cytokines at 20 ng/ml: IL-3 (R&D Systems, 203-IL-010), IL-6 (R&D Systems, 206-IL-025/CF), GM-CSF (StemCell Technologies, 78,015.1), and TPO (StemCell Technologies, 78,210). Samples were included solely based on availability, and the number of downstream assays was determined by the number of viable cells recovered. Prepared blasts were subsequently subjected to drug synergy assays, as described below.

### Compound preparation

All compounds were dissolved in either dimethyl sulfoxide (DMSO; cat. No. D8418, Sigma-Aldrich) or phosphate-buffered saline (PBS; cat. No. 14,190,144, Thermo Fisher Scientific), and stock solutions were prepared at concentrations ranging from 200 nM to 100 mM.The following compounds were used: cytarabine (ara-C; cat. No. 4520), disulfiram (DIS; cat. No. 3807), doxorubicin (DOXO; cat. No. 2252), fludarabine (F-ara-A; cat. No. 3495), nelarabine (cat. No. 6359), and ribavirin (RBV; cat. No. 4501) from Tocris; mycophenolic acid (MPA; cat. No. 1505), cladribine (2-CdA; cat. No. 5292), clofarabine (Cl-F-ara-A; cat. No. 2600) from Sigma-Aldrich; bredinin aglycone (BAg; cat. No. HY-106048) and venetoclax (VEN; cat. No. HY-15531) from MedChemExpress. All stock and working solutions were stored at −20°C.

### Proliferation inhibition and drug synergy assay

Single-agent and combination treatments were performed in 384-well plates (cat. No. 3570, Corning). Compounds were dispensed using a D300e Digital Dispenser (Tecan) with the Synergy Wizard in D300e Control Software. Final DMSO concentration was normalized to ≤1% in all wells.

For combination studies, single-agent dose-response curves were first generated to define appropriate concentration ranges. Prepared plates were either used immediately or stored at −20°C and equilibrated to room temperature for 30 min before cell plating.

Cells were seeded at 7500 cells per well in 25 µL medium, centrifuged briefly, and incubated for 72 h or 144 h at 37°C in a humidified incubator with 5% CO_2_. After incubation, plates were brought to room temperature for 30 min before the addition of 25 µL CellTiter-Glo® reagent (cat. No. G7573, Promega). Plates were shaken for 2 min, incubated for 10 min, and luminescence was measured using the Infinite® M PLEX reader (Tecan).Dose – response curves were fitted using a four-parameter logistic model in GraphPad Prism 10. Data were normalized to DMSO-only (negative control) and 100 µM doxorubicin (positive control). Normalized data were exported to Excel and analyzed using the SynergyFinder web tool [35]. Synergy scores were calculated using the Zero Interaction Potency (ZIP) model, Bliss independence, Loewe additivity, and Highest Single Agent (HSA), with scores > 10 indicating synergy, between −10 and 10 indicating additivity, and < −10 indicating antagonism [15]. Other cutoffs for synergistic and antagonistic drug interactions have been defined as > 5 and < −5, respectively [22]. ZIP scores reported correspond to the most synergistic area predicted by the model. For clarity and consistency in the main figures, only ZIP scores are presented, as this model integrates both potency and efficacy changes across the dose – response matrix, providing a robust estimation of the most synergistic interaction regions. For patient samples, MPA was tested at two concentrations and RBV at a single concentration due to limitations in available patient material, preventing the generation of full dose – response matrices. Consequently, synergy analysis was not performed for ara-C + RBV, and the scores obtained for ara-C + MPA should be considered indicative. Complete analyses for all four models, including dose – response plots and summary tables, are provided in Supplementary Figure S7 and Supplementary Table S2.

### Nucleotide measurements by HPLC

Cells were treated with the indicated compounds or vehicle (PBS) control for 24 h. Subsequently, cells were harvested, transferred to 50 mL tubes, and centrifuged at 500 × g for 3 min at 4°C. Cell pellets were washed in 20 mL ice-cold TBS (cat. No. 1,706,435, Bio-Rad), centrifuged again, and resuspended in 550 µL ice-cold lysis solution containing 15% trichloroacetic acid (TCA) (cat. No. T6499-100 G, Sigma-Aldrich) and 30 mM MgCl_2_. Samples were transferred to pre-chilled Eppendorf tubes, snap-frozen in liquid nitrogen, and stored at −80°C until nucleotide extraction and analysis.

Nucleotide pools were extracted and quantified as previously described [36], with minor modifications. Briefly, cells were lysed by vortex-mixing in ice-cold lysis solution using an Intelli-Mixer at 4°C for 10 min. Lysates were then centrifuged at 14,000 rpm for 3 min at 4°C. The resulting supernatant was neutralized twice by mixing with an equal volume of ice-cold dichloromethane – trioctylamine solution, followed by vortexing for 20 seconds and centrifugation at 14,000 rpm for 1 minute at 4°C.

Aqueous phase volume of 500 µL was subsequently adjusted to pH 8.9 using 1 M ammonium carbonate and applied to boronate affinity columns (Affi-Gel Boronate Gel, Bio-Rad). Elution was carried out with a solution containing 50 mM ammonium carbonate (pH 8.9) and 15 mM MgCl_2_ to separate dNTPs and ara-CTP from NTPs.

Eluates containing dNTPs and ara-nucleotides were adjusted to pH 3.4 and analyzed by HPLC with UV detection using a Partisphere SAX column (125 mm × 4.6 mm, 5 μm; Hichrom, UK). NTPs were directly analyzed from 25 µL aliquots of the aqueous phase, also adjusted to pH 3.4.

### Enzyme-coupled SAMHD1 dilution-jump ara-CTPase assay

GTP (cat. No. NU-1012) and ara-CTP (cat. No. NU-117) were obtained from Jena Bioscience. dATP (cat. No. 27–1850-04) and dGTP (cat. 27–1870-04) were obtained from GE Healthcare.

Recombinant His-tagged human SAMHD1 and *S. cerevisiae* PPX1 were expressed and purified by the Karolinska Institutet Protein Science Facility. The PPX1 plasmid (pScPPX2) was a gift from Michael Gray & Ursula Jakob (Addgene plasmid #112877; http://n2t.net/addgene:112877; RRID:Addgene_112877).

An enzyme-coupled dilution-jump assay is used to measure guanine nucleotide-dependent SAMHD1 hydrolase activity toward the triphosphate metabolite of cytarabine, ara-CTP. In a pre-jump oligomerisation step, SAMHD1 (20 µM) was combined with guanine nucleotide activators (GTP or dGTP, final concentration 100 µM), either in the presence or absence of allosteric site 2 activator dATP (final concentration 200 µM). After one minute, the oligomerisation mix was diluted 100-fold into buffer containing coupled enzyme PPX1 (final concentration 0.01 µM) and ara-CTP (final concentration 200 µM). See Supplementary Figure S6A for a schematic representation of the assay setup. Post-jump reactions were incubated for 20 minutes at room temperature, during which ara-CTP substrate is hydrolyzed to ara-C and inorganic triphosphate, which is further broken down into inorganic mono- and diphosphate by PPX1. Following the incubation, EDTA stop solution was added (final concentration: 3.95 mM). Following a 20-minute incubation with malachite green solution (final concentrations: 0.5 mM malachite green, 2.58 mM ammonium molybdate, 0.0036% Tween-20), the reaction progress can be quantified, as the complex formed between malachite green, inorganic phosphate and molybdate results in a change in absorbance at 630 nm. Absorbance measurements were performed with a Hidex Sense microplate reader (Hidex Oy) and the linear range of the assay determined with a sodium phosphate standard curve. All reactions took place in a 25 mM Tris-Acetate buffer (pH 8.0) containing 40 mM NaCl, 1 mM MgCl_2_, 0.005% Tween-20 and 0.3 mM TCEP in 384 microwell plates at a final volume of 25 µL.

### Protein detection by Western blot

Cells were collected at a density of approximately 2 × 10 [6] per sample, washed twice with phosphate-buffered saline (PBS; ThermoFisher, cat. No. 14,190,144) and lysed on ice for 1 h in radioimmunoprecipitation assay (RIPA) buffer (ThermoFisher, 89,900) supplemented with phosphatase (ThermoFisher, cat. No. 78,426) and protease inhibitors (Fisher Scientific, cat. No. 50–100-3270) at 1:100 and 1:7 dilutions, respectively. Lysates were vortexed intermittently every 10 min, followed by centrifugation at 14,000 × g for 20 min at 4°C. Supernatants containing soluble proteins were collected and stored at −20°C.

Protein concentrations were determined using the bicinchoninic acid (BCA) assay (ThermoFisher, cat. No. 23,225). Samples and standards were incubated in a 96-well plate (Sarstedt, cat. No. 83.3924.500) at 37°C for 30 min, and absorbance was measured at 562 nm using an Infinite® M PLEX plate reader (Tecan). Protein amounts were normalized to 10–15 µg per sample in a final volume of 20–30 µL.

Samples were mixed with Laemmli buffer (Bio-Rad, cat. No. 1,610,747) containing dithiothreitol (DTT; Thermo Scientific, cat. No. R0861), denatured at 95°C for 5 min, and loaded onto 10% Mini-PROTEAN® TGX Stain-Free™ Protein Gels (Bio-Rad, cat. No. 4,568,033/4568036) alongside a molecular weight ladder (Thermo Scientific, cat. No. 26,619). Electrophoresis was performed at 130–150 V for 60–90 min.

Proteins were transferred to nitrocellulose membranes using the Bio-Rad wet transfer system according to the manufacturer’s instructions. Transfer stacks were pre-soaked in cold transfer buffer (Bio-Rad, cat. No. 10,026,938) and assembled carefully to avoid air bubbles. Membranes were blocked in 5% nonfat milk in Tris-buffered saline with 0.1% Tween-20 (TBS-T; Sigma-Aldrich, cat. No. P1317-1 L) for 1–2 h at room temperature with gentle agitation.

Membranes were incubated overnight at 4°C with the following primary antibodies diluted in 5% milk/TBS-T: anti-SAMHD1 (rabbit, 1:1000, Fortis, cat. No. A303-691A), anti-IMPDH1 (rabbit, 1:3000, Proteintech, cat. No. 22,092–1-AP), anti-IMPDH2 (mouse, 1:5000, Proteintech, cat. No. 67,663–1-Ig), and anti-GAPDH (mouse, 1:5000, ThermoFisher, cat. No. 39–8600). Of note, both IMPDH antibodies have been validated to be isoform-specific by the manufacturer. The following day, membranes were washed three times for 15 min in TBS-T and incubated with HRP-conjugated secondary antibodies for 1 h at room temperature: anti-rabbit IgG (1:1000, Cell Signaling Technology, cat. No. 7074S) and anti-mouse IgG (1:5000, Cell Signaling Technology, cat. No. 7076P2).

After three additional washes in TBS-T, chemiluminescent signals were developed using ECL substrate (ThermoFisher, cat. No. 32,106) and visualized with a ChemiDoc™ Imaging System (Bio-Rad) using automatic exposure settings. Colourimetric and composite images were acquired to confirm transfer efficiency and protein loading. Unedited blots can be found in Supplementary Figure S8.

### Statistical analysis

Statistical analyses were performed using GraphPad Prism version 10 (GraphPad Software, San Diego, CA). Intracellular nucleotide and active metabolite levels, including dNTPs, NTPs, and ara-CTP, were quantified in three biological replicates, each comprising three technical replicates. ara-CTP values were normalized to CTP and then to ara-C alone within the same condition, whereas dNTP and NTP levels were normalized to CTP and subsequently to PBS-treated controls. Normality was assessed using the Shapiro – Wilk test. Comparisons of metabolite levels between PBS and treatment conditions (MPA, ara-C, ara-C + MPA) in THP-1 wild-type and knockout cells were performed using unpaired two-tailed t-tests. ara-CTP analysis was restricted to conditions involving ara-C and ara-C + MPA. Although the PBS control group was reused across comparisons, no correction for multiple comparisons was applied, as all comparisons were pre-specified, biologically distinct, and limited in number.

For IC_5__0_ fold-reduction analyses, unpaired two-tailed t-tests were applied when three biological replicates were available, each with two to three technical replicates. In experiments with only two biological replicates, the mean is shown, but no statistical testing was performed due to insufficient power. Where a third replicate was available but conducted with slightly different concentration ranges, these data were excluded from the mean yet showed consistent trends. Experiments with only one biological replicate are presented for illustrative purposes only, without statistical testing.

Unpaired two-tailed t-tests were also used to compare ZIP synergy scores between treatments. These analyses provided limited additional insight: most comparisons did not reach statistical significance, and when differences were detected, they were confined to the two cell lines with the most divergent ZIP values. Interpretation of combination effects is therefore based primarily on synergy plots.

Finally, for IC_5__0_ values analyzed individually across multiple cell lines, a Kruskal-Wallis test was performed to account for cases with low replicate numbers and datasets that did not pass the Shapiro-Wilk normality test. Although significant differences were detected between some cell lines, these results were not illustrated, as they did not provide additional biological insight beyond fold-reduction analyses and did not alter the overall interpretation of drug sensitivity.

## Supplementary Material

Supplemental Material
