# Supplementary material for: Targeting IMPDH to inhibit SAMHD1 in KMT2A-rearranged leukaemia
Source: Cell Cycle. 2025 Dec 15;25(1):1–19. doi: 10.1080/15384101.2025.2601796 (PMC12915856; doi:10.1080/15384101.2025.2601796)
Supplement: Supplemental Material [file KCCY_A_2601796_SM0380.docx]

**Supplementary Information for Klootsema et al.**

**Supplementary Table 1. Overview of cell line characteristics**

| **Cell line** | ***KMT2A* status** | **SAMHD1 status** | **Type of leukaemia** |
| --- | --- | --- | --- |
| **ALL-PO** | *KMT2A* wild-type | Proficient | B acute lymphoblastic leukaemia (childhood) |
| **HL-60 SAMHD1^+/+^** | *KMT2A* wild-type | Proficient | Acute myeloid leukaemia (adult) |
| **HL-60 SAMHD1^-/-^** | *KMT2A* wild-type | Deficient (SAMHD1^-/-^) | Acute myeloid leukaemia (adult) |
| **KASUMI-1** | *KMT2A* wild-type | Proficient | Acute myeloid leukaemia (adult) |
| **MOLT-16 SAMHD1^+/+^** | *KMT2A* wild-type | Proficient | T-cell acute lymphoblastic leukaemia (childhood) |
| **MOLT-16 SAMHD1^-/-^** | *KMT2A* wild-type | Deficient (SAMHD1^-/-^) | T-cell acute lymphoblastic leukaemia (childhood) |
| **OCI-AML3** | *KMT2A* wild-type | Proficient | Acute myeloid leukaemia (adult) |
| **SU223-T3** | *KMT2A* wild-type | Proficient | Induced pluripotent stem cell derived from human AML cells (adult) |
| **ML-2** | *KMT2A*–*MLLT4* fusion (*KMT2A*-rearranged) | Proficient | Acute myeloid leukaemia (adult) |
| **MONO-MAC-6** | *KMT2A*–*MLLT3* fusion (*KMT2A*-rearranged) | Proficient | Acute monocytic leukaemia (adult) |
| **MV4-11** | *KMT2A*–*AFF1* fusion (*KMT2A*-rearranged) | Proficient | Acute monocytic leukaemia (childhood) |
| **NOMO-1** | *KMT2A*–*MLLT3* fusion (*KMT2A*-rearranged) | Proficient | \| Acute myeloid leukaemia (adult) \| \| --- \|  \|  \| \| --- \| |
| **RS4;11** | *KMT2A*–*AFF1* fusion (*KMT2A*-rearranged) | Proficient | B-cell acute lymphoblastic leukaemia (adult) |
| **SEM** | *KMT2A*–*AFF1* fusion (*KMT2A*-rearranged) | Proficient | B-cell acute lymphoblastic leukaemia (childhood) |
| **SUPT-11 SAMHD1^+/+^** | *KMT2A*–*MLLT10* fusion (*KMT2A*-rearranged) | Proficient | T-cell acute lymphoblastic leukaemia (adult) |
| **SUPT-11 SAMHD1^-/-^** | *KMT2A*–*MLLT10* fusion (*KMT2A*-rearranged) | Deficient (SAMHD1^-/-^) | T-cell acute lymphoblastic leukaemia (adult) |
| **SU223-B3** | *KMT2A*–*MLLT3* fusion (*KMT2A*-rearranged) | Proficient | Induced pluripotent stem cell derived from human AML cells (adult) |
| **THP-1 SAMHD1^+/+^** | *KMT2A*–*MLLT3* fusion (*KMT2A*-rearranged) | Proficient | Acute monocytic leukaemia (childhood) |
| **THP-1 SAMHD1^-/-^** | *KMT2A*–*MLLT3* fusion (*KMT2A*-rearranged) | Deficient (SAMHD1^-/-^) | Acute monocytic leukaemia (childhood) |

**Supplementary Table 2.** Numerical synergy scores for multiple drug combinations across cell types and predictive models

| **Drug Combination** | **Sample name** | **Sample type** | **ZIP** | **Bliss** | **Loewe** | **HSA** |
| --- | --- | --- | --- | --- | --- | --- |
| **Ara-C + MPA** | **ALG24_052** | Patient-derived leukeamia cells | -0,83 | -0,55 | 0,43 | 5,33 |
|  | **ALG24_057** | Patient-derived leukeamia cells | -0,62 | -0,60 | 0,20 | 3,17 |
|  | **ALG25_013** | Patient-derived leukeamia cells | 1,52 | 0,49 | -0,23 | 8,58 |
|  | **ALG25_010** | Patient-derived leukeamia cells | 1,06 | 0,70 | -0,22 | 7,75 |
|  | **ALG25_015** | Patient-derived leukeamia cells | -0,03 | -0,32 | 0,48 | 6,78 |
|  | **ALG25_43** | Patient-derived leukeamia cells | 4,35 | 4,24 | -0,03 | 7,28 |
|  | **ALL-PO** | B-ALL cell line | 1,00 ± 1,39 | 0,47 ± 1,54 | 5,55 ± 2,72 | 3,13 ± 0,47 |
|  | **HL-60 SAMHD1^+/+^** | AML cell line | 0,44 ± 0,59 | 0,37 ± 0,71 | 2,98 ± 1,30 | 10,46 ± 0,99 |
|  | **HL-60 SAMHD1^-/-^** | AML cell line | 0,31 ± 0,54 | 0,04 ± 0,36 | -0,32 ± 0,31 | 2,78 ± 1,90 |
|  | **KASUMI-1** | AML cell line | 0,04 ± 0,84 | 0,25 ± 0,95 | 7,04 ± 2,24 | 2,40 ±1,16 |
|  | **ML-2** | AML cell line | 0,00 ± 0,31 | 0,01 ± 0,27 | 0,29 ± 0,32 | 7,18 ± 0,37 |
|  | **MONO-MAC-6** | AML cell line | -0,26 ± 0,18 | 0,88 ± 0,85 | 1,85 ± 2,78 | 4,96 ± 1,95 |
|  | **MV4-11** | AML cell line | 0,57 ± 0,68 | 0,59 ± 0,49 | 3,60 ± 1,93 | 3,60 ± 1,18 |
|  | **NOMO-1** | AML cell line | 1,70 ± 0,57 | 2,61 ± 0,85 | 3,91 ± 2,46 | 6,53 ± 2,45 |
|  | **OCI-AML3** | AML cell line | 1,52 ± 0,92 | 0,19 ± 0,88 | 3,41 ± 2,93 | 3,10 ± 0,81 |
|  | **RS4;11** | B-ALL cell line | 1,71 ± 1,77 | 0,69 ± 0,83 | 12,73 ± 12,86 | 5,89 ± 0,17 |
|  | **SEM** | B-ALL cell line | 0,89 ± 0,65 | 0,83 ± 0,81 | 2,97 ± 0,30 | 4,99 ±0,93 |
|  | **SU223-B3** | iPSCs | -0,02 | 0,01 | -0,07 | 3,49 |
|  | **SU223-T3** | iPSCs | 0,12 | -0,21 | 1,26 | 10,34 |
|  | **THP-1 SAMHD1^+/+^** | AML cell line | 12,8 ± 2,61 | 13,46 ± 2,37 | 16,78 ± 0,61 | 21,27 ± 3,51 |
|  | **THP-1 SAMHD1^-/-^** | AML cell line | 0,52 ± 0,94 | 0,25 ± 0,46 | 0,72 ± 1,53 | 3,06 ± 2,93 |
|  |  | | | | | |
| **Ara-C + RBV** | **ALL-PO** | B-ALL cell line | 1,11 ± 0,47 | 0,38 ± 0,90 | 1,98 ± 0,08 | 4,05 ± 0,26 |
|  | **HL-60 SAMHD1^+/+^** | AML cell line | 0,52 ± 0,79 | -0,09 ± 0,51 | 2,43 ± 4,25 | 3,74 ± 2,35 |
|  | **HL-60 SAMHD1^-/-^** | AML cell line | 0,49 ± 0,69 | 0,01 ± 0,37 | -0,15 ± 0,20 | 2,12 ± 1,49 |
|  | **KASUMI-1** | AML cell line | -1,84 ± 0,95 | -3,70 ± 0,29 | 4,85 ± 1,05 | 7,18 ± 1,29 |
|  | **ML-2** | AML cell line | 6,00 ± 3,33 | 5,92 ± 2,76 | 13,35 ± 7,23 | 13,75 ±4,00 |
|  | **MONO-MAC-6** | AML cell line | -1,14 ± 0,14 | -1,05 ± 0,21 | 1,83 ± 1,73 | 5,15 ± 2,73 |
|  | **MV4-11** | AML cell line | 0,33 ± 0,12 | 0,30 ± 0,16 | 41,17 ± 1,61 | 4,17 ± 0,44 |
|  | **NOMO-1** | AML cell line | 2,10 ± 0,18 | 2,14 ± 0,30 | 5,25 ± 0,01 | 10,53 ± 0,22 |
|  | **OCI-AML3** | AML cell line | -0,21 ± 0,66 | -0,81 ± 0,33 | 4,54 ± 0,16 | 5,54 ± 0,76 |
|  | **RS4;11** | B-ALL cell line | 0,08 ± 0,42 | -0,28 ± 0,47 | 6,58 ± 7,13 | 6,20 ± 1,16 |
|  | **SEM** | B-ALL cell line | -0,34 ± 0,30 | -0,63 ± 1,29 | 2,30 ± 3,06 | 5,44 ± 4,00 |
|  | **THP-1 SAMHD1^+/+^** | AML cell line | 3,00 ± 4,15 | 2,27 ± 4,32 | 3,91 ± 2,95 | 8,22 ± 4,45 |
|  | **THP-1 SAMHD1^-/-^** | AML cell line | 0,53 ± 0,49 | 0,37 ± 0,57 | 1,06 ± 1,66 | 3,90 ± 1,25 |
|  |  | | | | | |
| **Ara-C + BAg** | **ALL-PO** | B-ALL cell line | 0,22 ± 1,63 | 0,15 ± 1,81 | 5,56 ± 1,62 | 3,84 ± 3,18 |
|  | **HL-60 SAMHD1^+/+^** | AML cell line | 0,47 ± 0,23 | 0,59 ± 0,81 | 0,48 ± 1,82 | 3,28 ± 2,15 |
|  | **HL-60 SAMHD1^-/-^** | AML cell line | 0,28 ± 0,42 | -1,38 ± 3,17 | 0,05 ± 0,26 | 1,64 ± 1,56 |
|  | **RS4;11** | B-ALL cell line | -0,37 ± 0,73 | 0,35 ± 0,95 | 4,03 ± 6,70 | 0,95 ± 1,46 |
|  | **SEM** | B-ALL cell line | 0,23 ± 0,72 | 0,88 ± 1,44 | -2,4 ± 2,67 | 2,38 ± 3,56 |
|  | **THP-1 SAMHD1^+/+^** | AML cell line | -2,46 ± 2,99 | -1,73 ± 2,35 | 2,39 ± 0,74 | 3,33 ± 0,93 |
|  | **THP-1 SAMHD1^-/-^** | AML cell line | -0,64 ± 0,83 | -1,08 ± 1,53 | 0,09 ± 1,98 | -0,10 ± 1,51 |
|  |  | | | | | |
| **Ara-C + DIS** | **HL-60 SAMHD1^+/+^** | AML cell line | 0,62 ± 0,47 | 0,72 ± 0,70 | 5,51 ± 7,28 | 6,05 ± 1,28 |
|  | **HL-60 SAMHD1^-/-^** | AML cell line | 0,14 ± 0,35 | -0,15 ± 0,10 | 5,74 ± 5,86 | 4,07 ± 3,11 |
|  | **THP-1 SAMHD1^+/+^** | AML cell line | -1,31 ± 0,91 | -1,10 ± 0,89 | 0,23 ± 0,87 | 4,64 ± 2,29 |
|  | **THP-1 SAMHD1^-/-^** | AML cell line | 0,17 ± 0,92 | 0,25 ± 0,88 | 1,96 ± 1,98 | 3,31 ± 4,46 |
|  |  | | | | | |
| **2-CdA + MPA** | **ML-2** | AML cell line | 2,01 ± 0,09 | 2,18 ± 0,10 | 4,04 ± 1,18 | 5,08 ± 1,24 |
|  | **THP-1 SAMHD1^+/+^** | AML cell line | 1,93 ± 1,90 | 2,78 ± 1,53 | -0,15 ± 1,18 | 10,74 ± 0,11 |
|  |  | | | | | |
| **2-CdA + RBV** | **ML-2** | AML cell line | 6,19 ± 0,52 | 6,43 ± 1,64 | 9,82 ± 5,10 | 12,81 ± 1,48 |
|  | **THP-1 SAMHD1^+/+^** | AML cell line | -1,13 ± 1.59 | -1,30 ± 0,03 | 10,28 ± 11,67 | 5,69 ± 1,48 |
|  |  | | | | | |
| **Cl-F-ara-A + MPA** | **ML-2** | AML cell line | 2,84 ± 0,16 | 3,20 ± 0,07 | 6,28 ± 0,30 | 4,86 ± 1,59 |
|  | **THP-1 SAMHD1^+/+^** | AML cell line | 4,06 ± 0,16 | 4,08 ± 0,32 | 1,49 ± 1,15 | 12,12 ± 0,45 |
|  |  | | | | | |
| **Cl-F-ara-A + RBV** | **ML-2** | AML cell line | 1,38 ± 1,24 | 2,39 ± 2,04 | 5,93 ± 0,06 | 4,15 ± 2,06 |
|  | **THP-1 SAMHD1^+/+^** | AML cell line | 3,07 ± 1,56 | 3,18 ± 1,58 | 4,12 ± 2,57 | 9,91 ± 2,67 |
|  |  | | | | | |
| **F-ara-A + MPA** | **ML-2** | AML cell line | 3,76 ± 1,45 | 3,83 ± 1,74 | 10,24 ± 1,15 | 8,10 ± 0,80 |
|  | **THP-1 SAMHD1^+/+^** | AML cell line | 8,64 ± 4,48 | 9,17 ± 4,42 | 18,72 ± 7,15 | 17,36 ± 2,40 |
|  |  | | | | | |
| **F-ara-A + RBV** | **ML-2** | AML cell line | 5,73 ± 4,22 | 5,83 ± 4,28 | 21,77 ± 1,42 | 9,56 ± 5,06 |
|  | **THP-1 SAMHD1^+/+^** | AML cell line | 8,27 ± 4.38 | 8,61 ± 3,38 | 23,92 ± 1,64 | 14,50 ± 3,61 |
|  |  | | | | | |
| **Nelarabine + MPA** | **MOLT-16 SAMHD1^+/+^** | T-ALL cell line | 0,52 | 0,83 | 19,68 | 1,20 |
|  | **MOLT-16 SAMHD1^-/-^** | T-ALL cell line | 4,09 | 4,93 | 10,21 | 6,03 |
|  | **SUPT-11 SAMHD1^+/+^** | T-ALL cell line | 1,47 ± 0,50 | 1,44 ± 0,30 | 5,71 ± 0,77 | 7,20 ± 2,24 |
|  | **SUPT-11 SAMHD1^-/-^** | T-ALL cell line | 1,59 ± 2,67 | 2,22 ± 3,74 | 4,36 ± 0,33 | 6,61 ± 4,15 |
|  |  | | | | | |
| **VEN + MPA** | **HL-60 SAMHD1^+/+^** | AML cell line | 2,60 ± 0,67 | 3,18 ± 0,25 | 3,14 ± 5,55 | 15,28 ± 3,30 |
|  | **HL-60 SAMHD1^-/-^** | AML cell line | 3,58 ± 1,62 | 3,46 ± 1,43 | 15,39 ± 9,49 | 15,89 ± 2,41 |
|  | **THP-1 SAMHD1^+/+^** | AML cell line | 18,06 ± 4,95 | 16,66 ± 4,82 | 21,90 ± 11.71 | 24,86 ± 7.07 |
|  | **THP-1 SAMHD1^-/-^** | AML cell line | 14,75 ± 2,66 | 3,42 ± 0,95 | 27,43 ± 4,45 | 29,33 ± 3,07 |

**Supplementary Table 3. Characteristics of adult primary patient samples**

| **Sample ID** | **Diagnosis** | **Gender** | ***KMT2A* status** | **Cytogenetics** | ***FLT3*** | ***NPM1*** | **Other mutations** |
| --- | --- | --- | --- | --- | --- | --- | --- |
| ALG24_052 | AML | Male | Partial tandem duplication (exon 2-10) | 46,XY,[20] | WT | WT | *ASXL1, IDH1, IDH2, RUNX1, SRSF2* |
| ALG24_057 | AML | Female | Partial tandem duplication (exon 2-8) | 46,XX,[20] | WT | WT | *IDH1, SRSF2, STAG2* |
| ALG25_013 | CMML | Male | Partial tandem duplication exon 2-8 | 46,XY,[21] | WT | WT | *ASXL1, IDH1, RUNX1* |
| ALG25_043 | Monocytic AML | Female | WT | 46,XX,t(9;11)(p21;23)[20] | p.Asp835Tyr | WT |  |
| ALG25_015 | AML | Female | WT | 46,XX,[20] | WT | p.Trp288Cysfs*12 | *PTPN11* |
| ALG25_010 | AML | Female | WT | 46,XX,inv(16)(p13q22)[11] | WT | WT | *KIT, KRAS, NRAS* |

AML: acute myeloid leukemia; CMML: chronic myelomonocytic leukemia; FLT3: fms-like tyrosine kinase 3; WT: wild type; NPM1: nucleophosmin

**
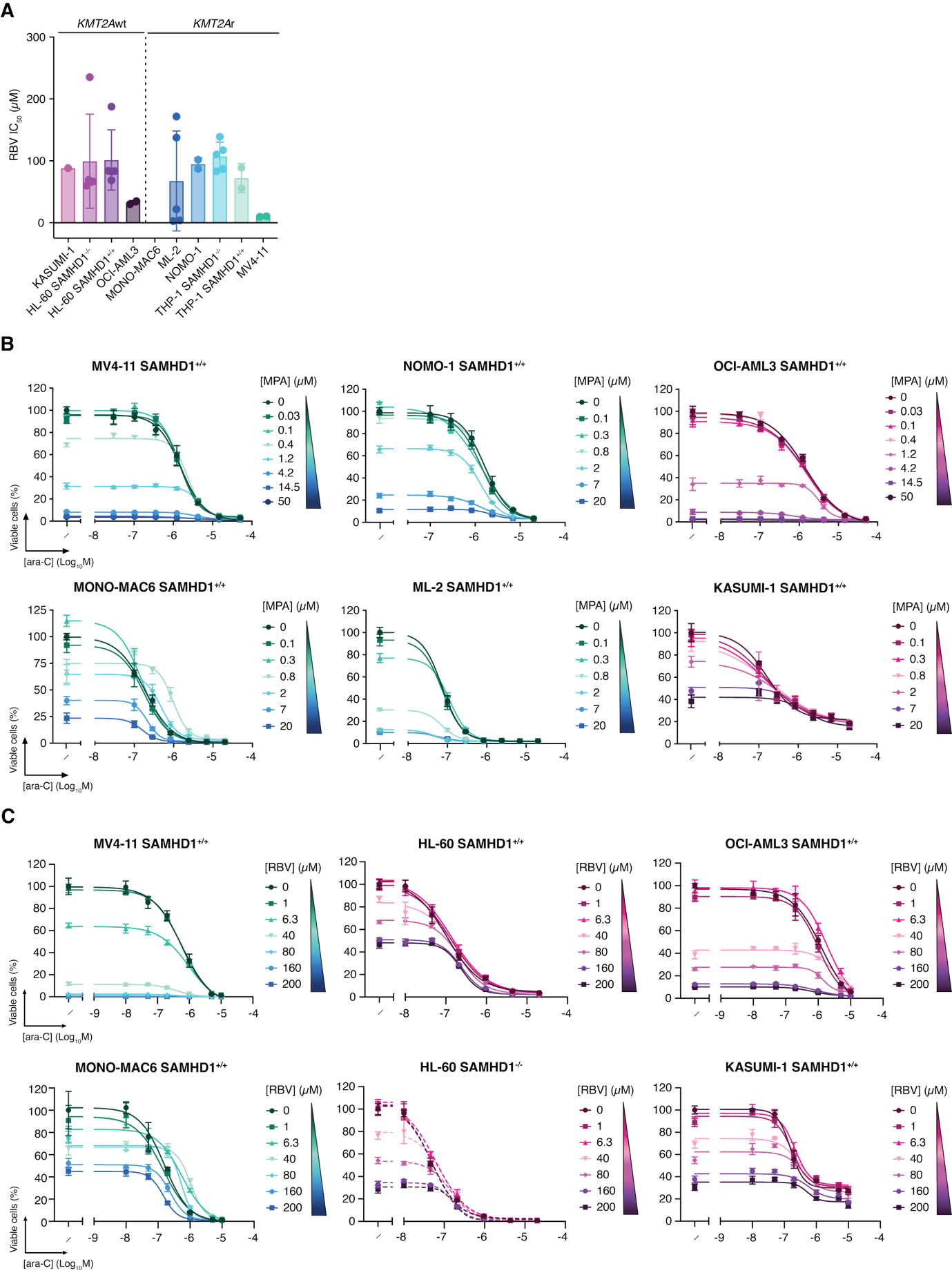
**

**Supplementary Figure 1: Combination of IMPDHi with ara-C in AML.**

**(A)** RBV IC₅₀ values across AML cell lines. Bars indicate mean from a minimum of two independent experiments, with error bars showing SD and points corresponding to individual experiments.

**(B, C)** Representative dose–response curves of ara-C combined with MPA (B) or RBV (C) in the indicated AML cell lines. Data are mean ± SD from one experiment representative of at least two biological replicates.

**Data information:** For (A) statistical significance for individual RBV IC₅₀ values across multiple cell lines was assessed using the Kruskal–Wallis test. Although significant differences were detected between some cell lines, these results are not shown, as they did not provide additional information beyond fold-reduction analyses (see Methods).

**
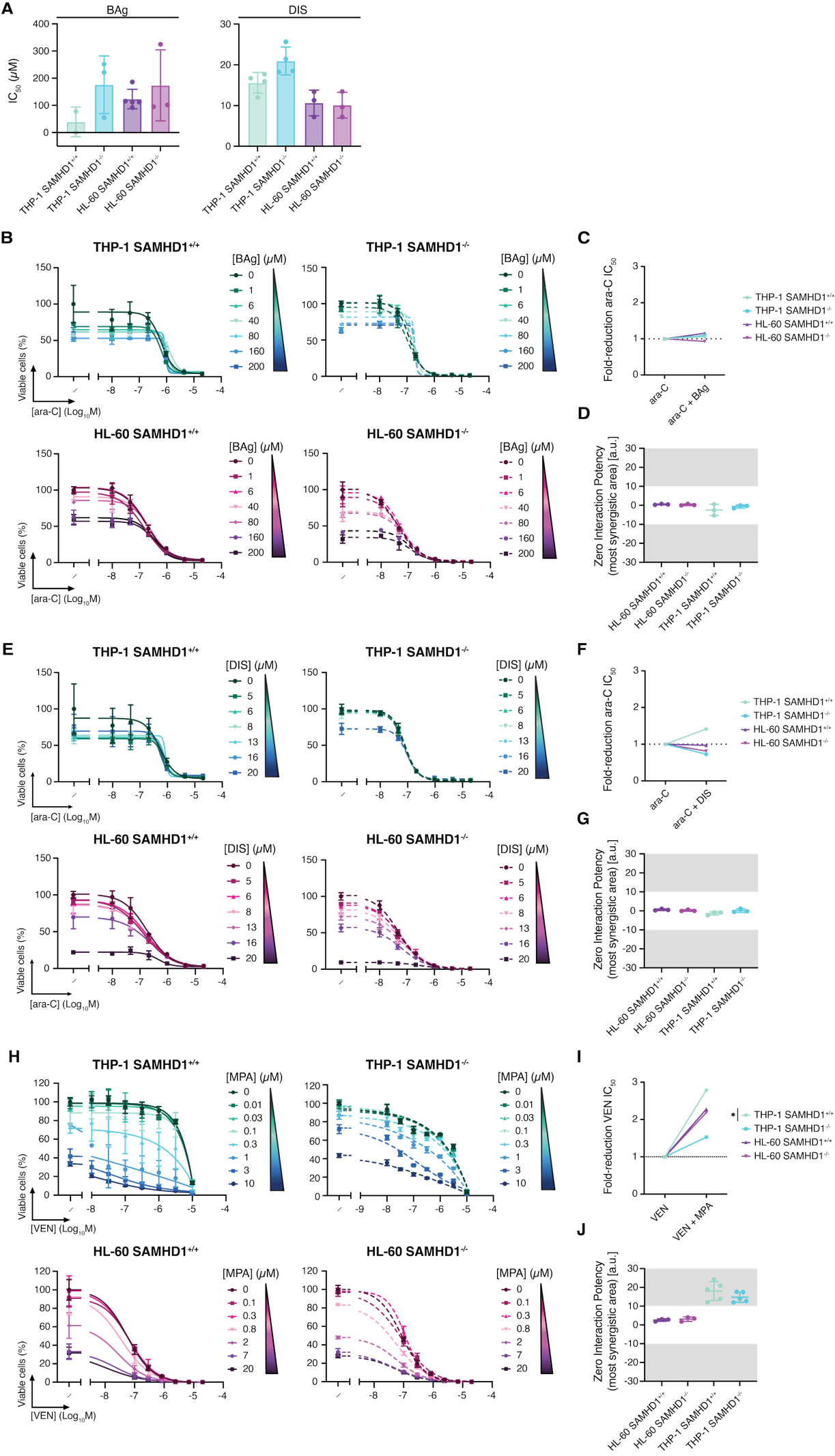
**

**Supplementary Figure 2: Combination of IMPDHi with ara-C in AML.**

**(A)** BAg and DIS IC₅₀ values in THP-1 and HL-60 both SAMHD1^+/+^ and SAMHD1^-/-^ cell lines. Bars indicate mean from a minimum of two independent experiments, with error bars showing SD and points corresponding to individual experiments.

**(B, E, H)** Representative dose–response curves of ara-C combined with (B) BAg or (E) DIS and (H) VEN combined with MPA in THP-1 and HL-60 cells with or without SAMHD1. Data are mean ± SD from one experiment representative of three biological replicates.

**(C, F, I)** Maximum fold-reduction in ara-C with (C) BAg or (F) DIS and (I) VEN IC₅₀ upon co-treatment with MPA based on three independent experiments. Only concentrations at which BAg, DIS or MPA alone reduced viability by ≤80% were included, to avoid confounding effects from cytotoxicity. Each dot represents the mean fold reduction in ara-C or VEN IC₅₀ across three independent experiments, based on the combination condition that yielded the greatest reduction. Concentrations of IMPDHi used for each condition were as follows: BAg - 1 µM for all cell lines (THP-1 and HL-60, SAMHD1^+/+^ and SAMHD1^-/-^); DIS - 16 µM for THP-1 SAMHD1^+/+^ and SAMHD1^-/-^, 5 µM for HL-60 SAMHD1^+/+^ and SAMHD1^-/-^; MPA - 0.1 µM for THP-1 SAMHD1^+/+^ and SAMHD1^-/-^, 7 µM for HL-60 SAMHD1^+/+^ and SAMHD1^-/-^.

**(D, G, J)** ZIP synergy scores for the most synergistic area for ara-C + (D) BAg, (G) DIS, and (K) VEN + MPA, shown as mean ± SD with individual values from three independent experiments.

**Data information:** For (A) individual IC₅₀ values compared across multiple cell lines, a Kruskal–Wallis test was performed; although some significant differences were observed, they are not shown, as they did not provide additional information beyond fold-reduction analyses (see Methods). For (C, D, F, G, I, J), statistical significance was assessed using unpaired two-tailed t-tests (*p < 0.05, **p < 0.01, ***p < 0.001, ****p < 0.0001); the absence of symbols indicates that statistical analysis was not significant or not performed (see Methods). Detailed results for each maximum fold-reduction in VEN IC_50_ are as follows: Fig S1I – VEN vs VEN + MPA: THP-1 SAMHD1^+/+^, *n* = 3, *P* = 0.0104, *t* = 4.547, *df* = 4.


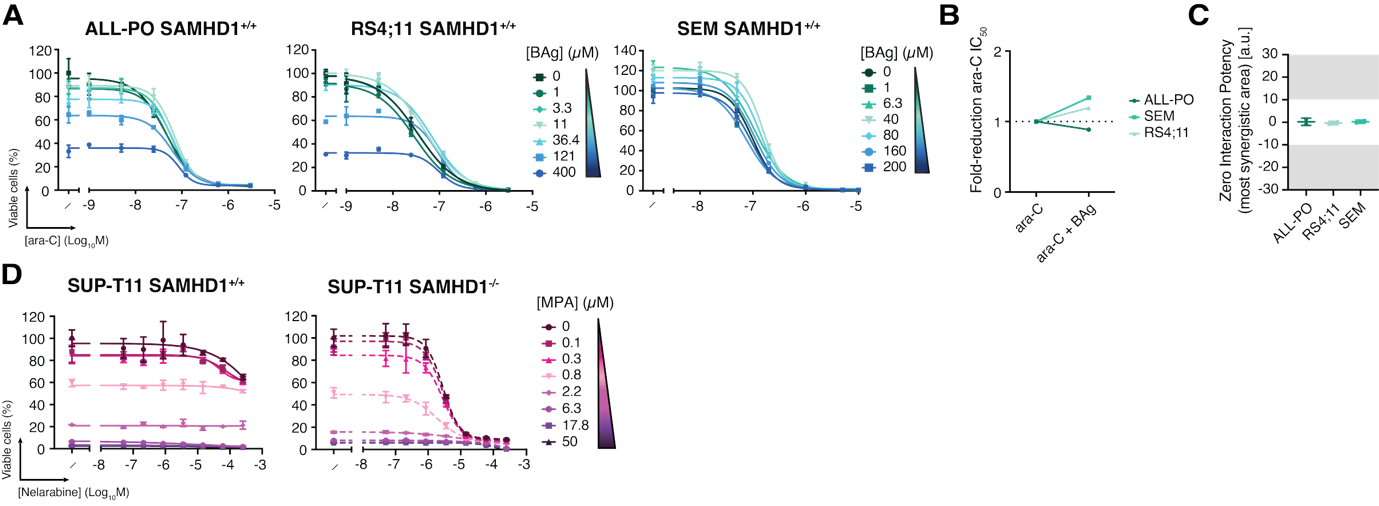


**Supplementary Figure 3: Lack of synergy between IMPDHi and ara-C in B-ALL and T-ALL models.**

**(A, D)** Representative dose–response curves of (A) ara-C combined with BAg in B-ALL cell lines (ALL-PO, RS4;11, SEM) or (D) nelarabine combined with MPA in T-ALL cell lines (SUP-T11 with or without SAMHD1). Data are mean ± SD from one experiment representative of two biological replicates.

**(B)** Maximum fold-reduction in ara-C IC₅₀ upon co-treatment with MPA in B-ALL cell lines (ALL-PO, RS4;11, SEM). Only concentrations at which ara-C alone reduced viability by ≤80% were included, to avoid confounding effects from cytotoxicity. Each dot represents the mean fold reduction in ara-C IC₅₀ across two independent experiments, based on the combination condition that yielded the greatest reduction. Fixed concentrations of BAg or MPA used in each ALL model were as follows: ara-C combined with BAg - ALL-PO, SEM, and RS4;11, 1 µM; ara-C or nelarabine combined with MPA - SUPT11 SAMHD1^-/-^, 0.8 µM; SUPT11 SAMHD1^+/+^ (data not consistently reproducible and therefore not represented).

**(C)** ZIP synergy scores for ara-C + BAg in B-ALL cell lines (ALL-PO, RS4;11, SEM), shown as mean ± SD with individual values from two independent experiments.

**Data information:** Statistical significance was assessed using unpaired two-tailed t-tests (*p < 0.05, **p < 0.01, ***p < 0.001, ****p < 0.0001); the absence of symbols indicates that statistical analysis was not significant or not performed (see Methods).


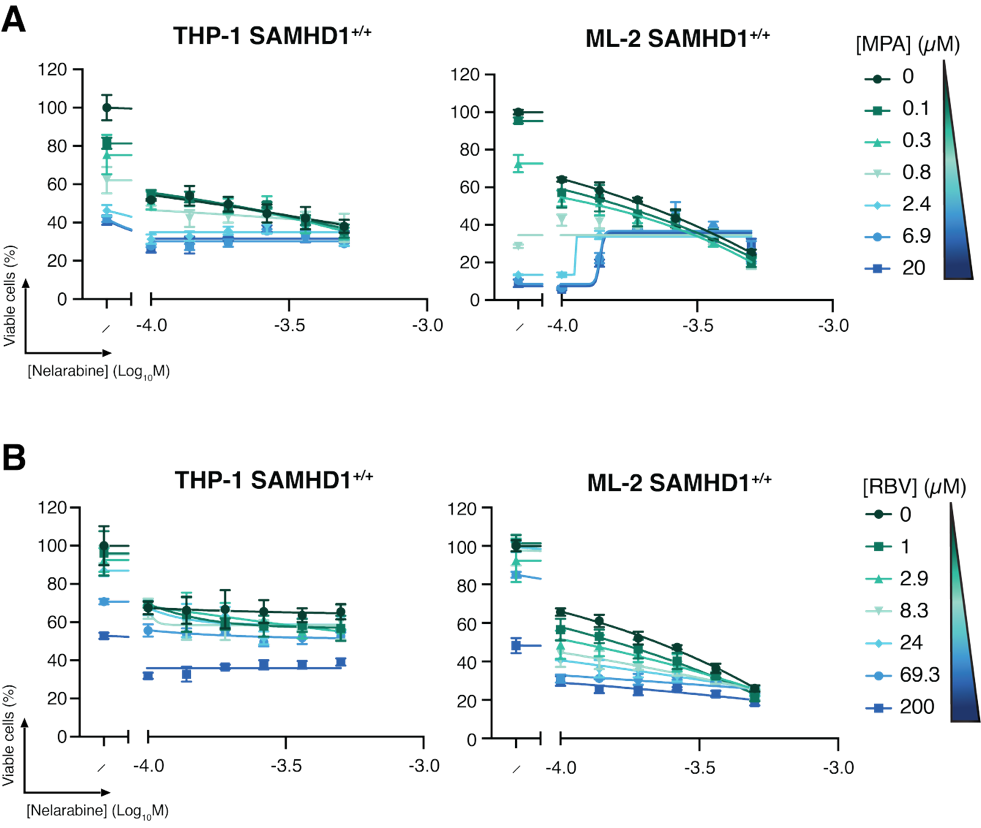


**Supplementary Figure 4: Limited nelarabine activity and its modulation by MPA in THP-1 SAMHD1^+/+^ and ML-2 cells.**

**(A, B)** Representative dose–response curves of nelarabine combined with either (A) MPA or (B) RBV in THP-1 SAMHD1^+/+^ and ML-2. Data are mean ± SD from one experiment representative of at least one biological replicates.


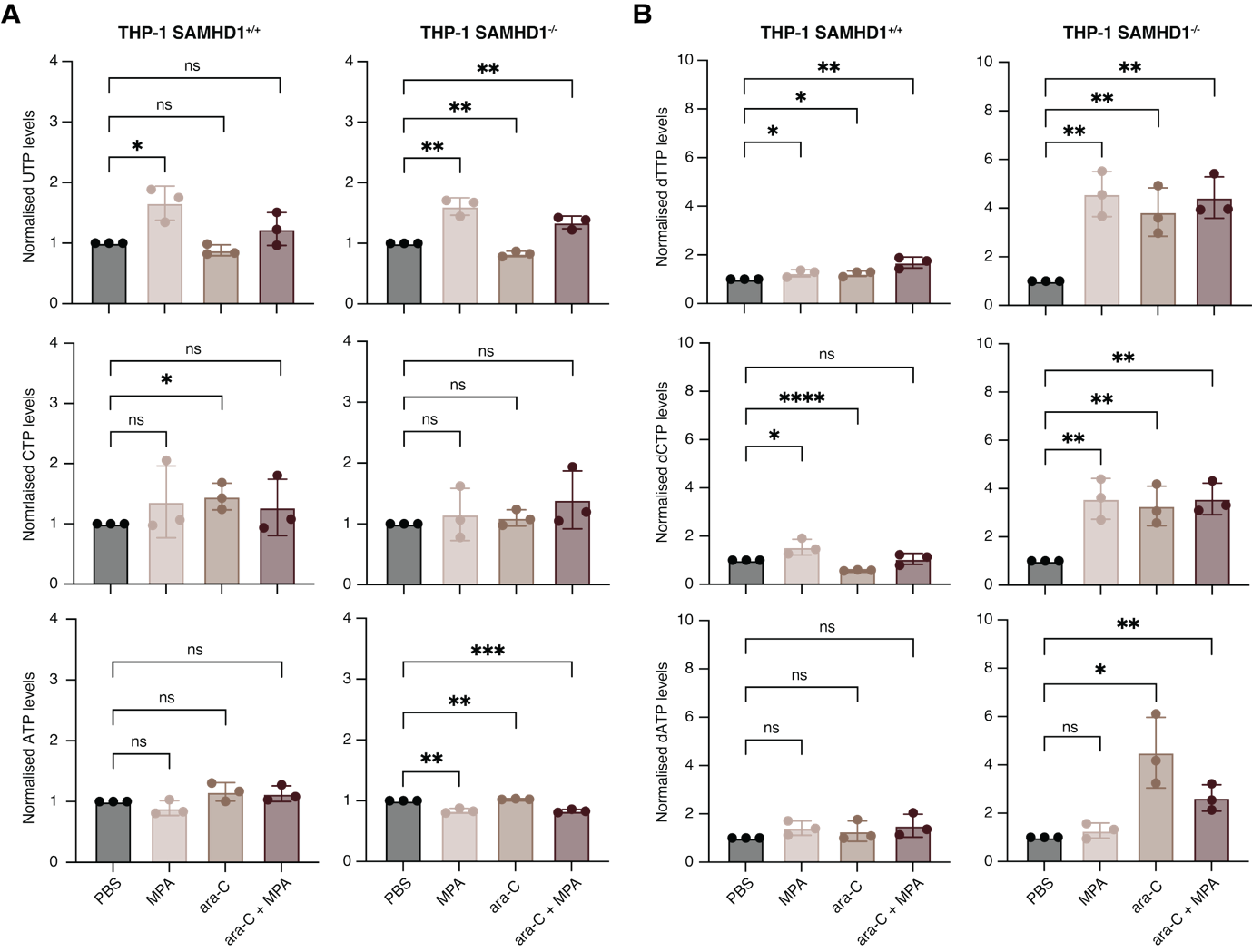


**Supplementary Figure 5: dNTP and NTP pools in THP-1 cells upon treatment.**

Intracellular (A) UTP, CTP and ATP levels and (B) dTTP, dCTP, and dATP levels were measured in THP-1 SAMHD1^+/+^ and SAMHD1^-/-^ cells after 24-hour treatment with PBS (vehicle), MPA (20µM), ara-C (500nM) or the combination of MPA (20µM) and ara-C (550nM). Metabolite levels are shown as values normalised to CTP and the PBS condition, representing relative levels per million cells. Data represent means ± SD of three independent experiments, with each dot representing one biological replicate.

**Data information:** For (A, B) statistical significance was assessed using unpaired two-tailed t-tests (*p < 0.05, **p < 0.01, ***p < 0.001, ****p < 0.0001); the absence of symbols indicates that statistical analysis was not significant or not performed (see Methods). Detailed results for each nucleotide and treatment in THP-1 wild-type (SAMHD1^+/+^) and knockout (SAMHD1^-/-^) cells are as follows: Fig EV2A - UTP (PBS vs MPA): THP-1 SAMHD1^+/+^, *n* = 3, *P* = 0.0153, *t* = 4.068, *df* = 4; THP-1 SAMHD1^-/-^, *n* = 3, *P* = 0.0018, *t* = 7.392*, df* = 4. UTP (PBS vs ara-C): THP-1 SAMHD1^+/+^, *n* = 3, *P* = 0.0853, *t* = 2.275, *df* = 4; THP-1 SAMHD1^-/-^, *n* = 3, *P* = 0.0024, *t* = 6.844*, df* = 4. UTP (PBS vs ara-C + MPA): THP-1 SAMHD1^+/+^; *n* = 3, *P* = 0.2111, *t* = 1.488, *df* = 4; THP-1 SAMHD1^-/-^, *n* = 3, *P* = 0.0048, *t* = 5.660*, df* = 4. CTP (PBS vs MPA): THP-1 SAMHD1^+/+^, *n* = 3, *P* = 0.3503, *t* = 1.057, *df* = 4; THP-1 SAMHD1^-/-^, *n* = 3, *P* = 0.5667, *t* = 0.6236*, df* = 4. CTP (PBS vs ara-C): THP-1 SAMHD1^+/+^, *n* = 3, *P* = 0.0242, *t* = 3.534, *df* = 4; THP-1 SAMHD1^-/-^, *n* = 3, *P* = 0.2678, *t* = 1.286*, df* = 4. CTP (PBS vs ara-C + MPA): THP-1 SAMHD1^+/+^, *n* = 3, *P* = 0.3693, *t* = 1.011, *df* = 4; THP-1 SAMHD1^-/-^, *n* = 3, *P* = 0.2254, *t* = 1.432*, df* = 4. ATP (PBS vs MPA): THP-1 SAMHD1^+/+^, *n* = 3, *P* = 0.1990, *t* = 1.537, *df* = 4; THP-1 SAMHD1^-/-^, *n* = 3, *P* = 0.0018, *t* = 7.366*, df* = 4. ATP (PBS vs ara-C): THP-1 SAMHD1^+/+^, *n* = 3, *P* = 0.1407, *t* = 1.833, *df* = 4; THP-1 SAMHD1^-/-^, *n* = 3, *P* = 0.0012, *t* = 8.168*, df* = 4. ATP (PBS vs ara-C + MPA): THP-1 SAMHD1^+/+^, *n* = 3, *P* = 0.1536, *t* = 1.758, *df* = 4; THP-1 SAMHD1^-/-^, *n* = 3, *P* = 0.0004, *t* = 10.97*, df* = 4. Fig EV2B - dTTP (PBS vs MPA): THP-1 SAMHD1^+/+^, *n* = 3, *P* = 0.0401, *t* = 2.996, *df* = 4; THP-1 SAMHD1^-/-^, *n* = 3, *P* = 0.0026, *t* = 6.697*, df* = 4. dTTP (PBS vs ara-C): THP-1 SAMHD1^+/+^, *n* = 3, *P* = 0.0214, *t* = 3.670, *df* = 4; THP-1 SAMHD1^-/-^, *n* = 3, *P* = 0.0078, *t* = 4.938*, df* = 4. dTTP (PBS vs ara-C + MPA): THP-1 SAMHD1^+/+^, *n* = 3, *P* = 0.0061, *t* = 5.303, *df* = 4; THP-1 SAMHD1^-/-^, *n* = 3, *P* = 0.0022, *t* = 7.004*, df* = 4. dCTP (PBS vs MPA): THP-1 SAMHD1^+/+^, *n* = 3, *P* = 0.0443, *t* = 2.895, *df* = 4; THP-1 SAMHD1^-/-^, *n* = 3, *P* = 0.0062, *t* = 5.276*, df* = 4. dCTP (PBS vs ara-C): THP-1 SAMHD1^+/+^, *n* = 3, *P* = < 0.0001, *t* = 32.36, *df* = 4; THP-1 SAMHD1^-/-^, *n* = 3, *P* = 0.0086, *t* = 4.811*, df* = 4. dCTP (PBS vs ara-C + MPA): THP-1 SAMHD1^+/+^, *n* = 3, *P* = 0.06977, *t* = 0.4175, *df* = 4; THP-1 SAMHD1^-/-^; *n* = 3, *P* = 0.0024, *t* = 6.819*, df* = 4. dATP (PBS vs MPA): THP-1 SAMHD1^+/+^, *n* = 3, *P* = 0.0744, *t* = 2.399, *df* = 4; THP-1 SAMHD1^-/-^, *n* = 3, *P* = 0.1961, *t* = 1.550*, df* = 4. dATP (PBS vs ara-C): THP-1 SAMHD1^+/+^, *n* = 3, *P* = 0.3082, *t* = 1.167, *df* = 4; THP-1 SAMHD1^-/-^, *n* = 3, *P* = 0.0142, *t* = 4.159*, df* = 4. dATP (PBS vs ara-C + MPA): THP-1 SAMHD1^+/+^, *n* = 3, *P* = 0.1350, *t* = 1.869, *df* = 4; THP-1 SAMHD1^-/-^, *n* = 3, *P* = 0.0066, *t* = 5.179*, df* = 4

**Supplementary Figure 6: Guanine nucleotides are required for ara-CTP hydrolysis by SAMHD1.**

**(A)** Schematic representation of the enzyme-coupled SAMHD1 dilution-jump ara-CTPase assay. SAMHD1 is pre-incubated with guanine nucleotide activators (negative control: only buffer), either in the absence (left side) or presence (right side) of additional allosteric site 2 activator dATP. The pre-jump mix is then diluted 100-times by addition of buffer containing coupled enzyme PPX1 and ara-CTP substrate.

**(B)** SAMHD1 ara-CTPase activity, normalised to the GTP (100 µM) + dATP (200 µM) pre-jump condition. Bars indicate mean of three independent experiments where dots represent mean values of technical duplicates per experiment. Error bars indicate SD.


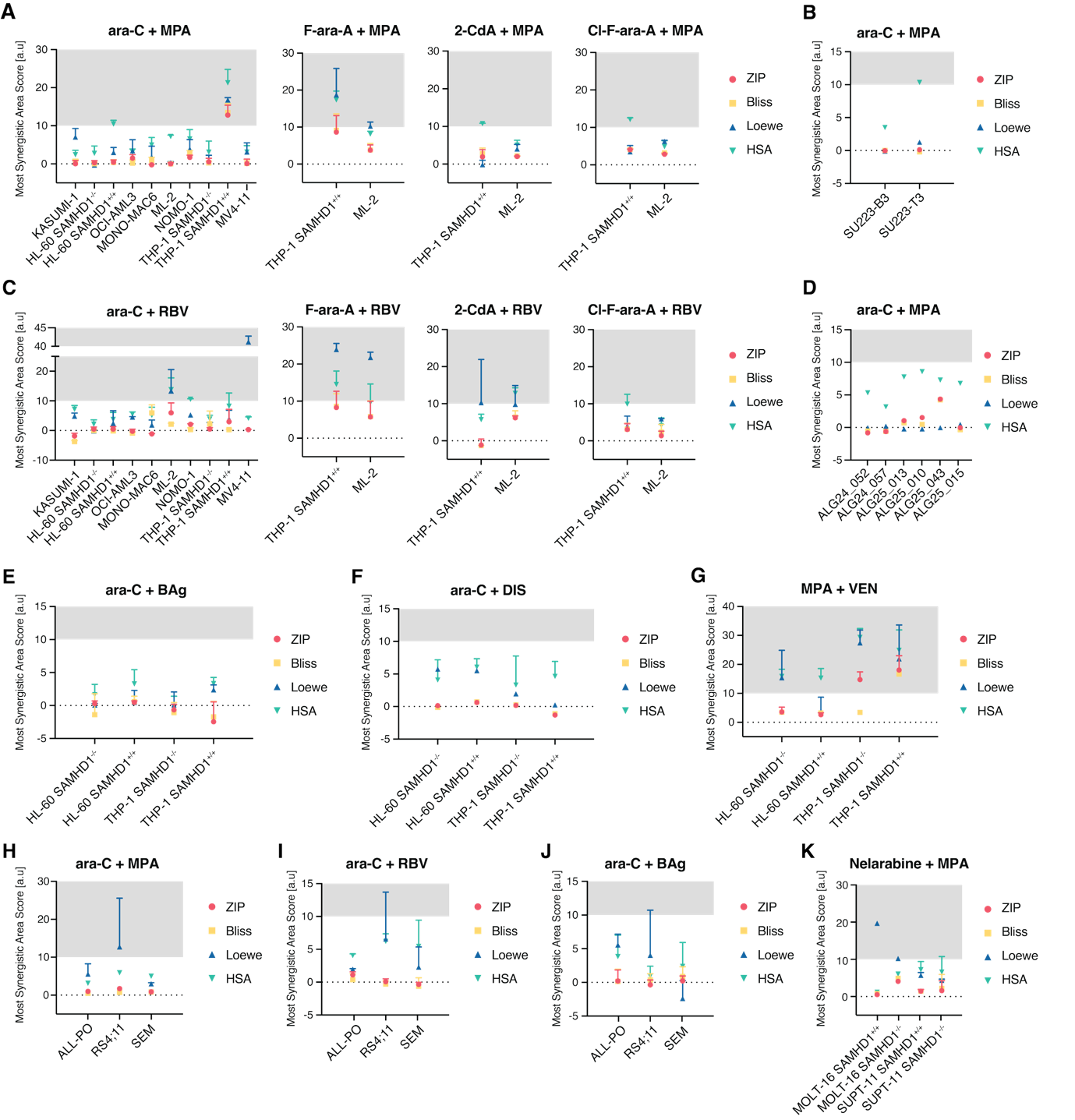


**Supplementary Figure 7: Synergy analysis of multiple drug combinations across cell lines, iPSCs, and patient-derived leukaemia samples.**

Synergy was calculated using four predictive models: ZIP, Bliss, Loewe, and HSA. Panels show the following combinations and sample types: **(A)** ara-C + MPA, F-ara-A + MPA, 2-CdA + MPA, Cl-F-ara-A + MPA in AML cell lines; **(B)** ara-C + MPA in iPSCs; **(C)** ara-C + RBV, F-ara-A + RBV, 2-CdA + RBV, Cl-F-ara-A + RBV in AML cell lines; **(D)** ara-C + MPA in patient-derived leukaemia cells; **(E)** ara-C + BAg in AML cell lines; **(F)** ara-C + DIS in AML cell lines; **(G)** MPA + VEN in AML cell lines; **(H)** ara-C + MPA in B-ALL cell lines; **(I)** ara-C + RBV in B-ALL cell lines; **(J)** ara-C + BAg in B-ALL cell lines; **(K)** nelarabine + MPA in T-ALL cell lines. Values are shown as mean ± SD for one or more experiments per combination, with each point representing the mean of the available experiments for that condition.

**
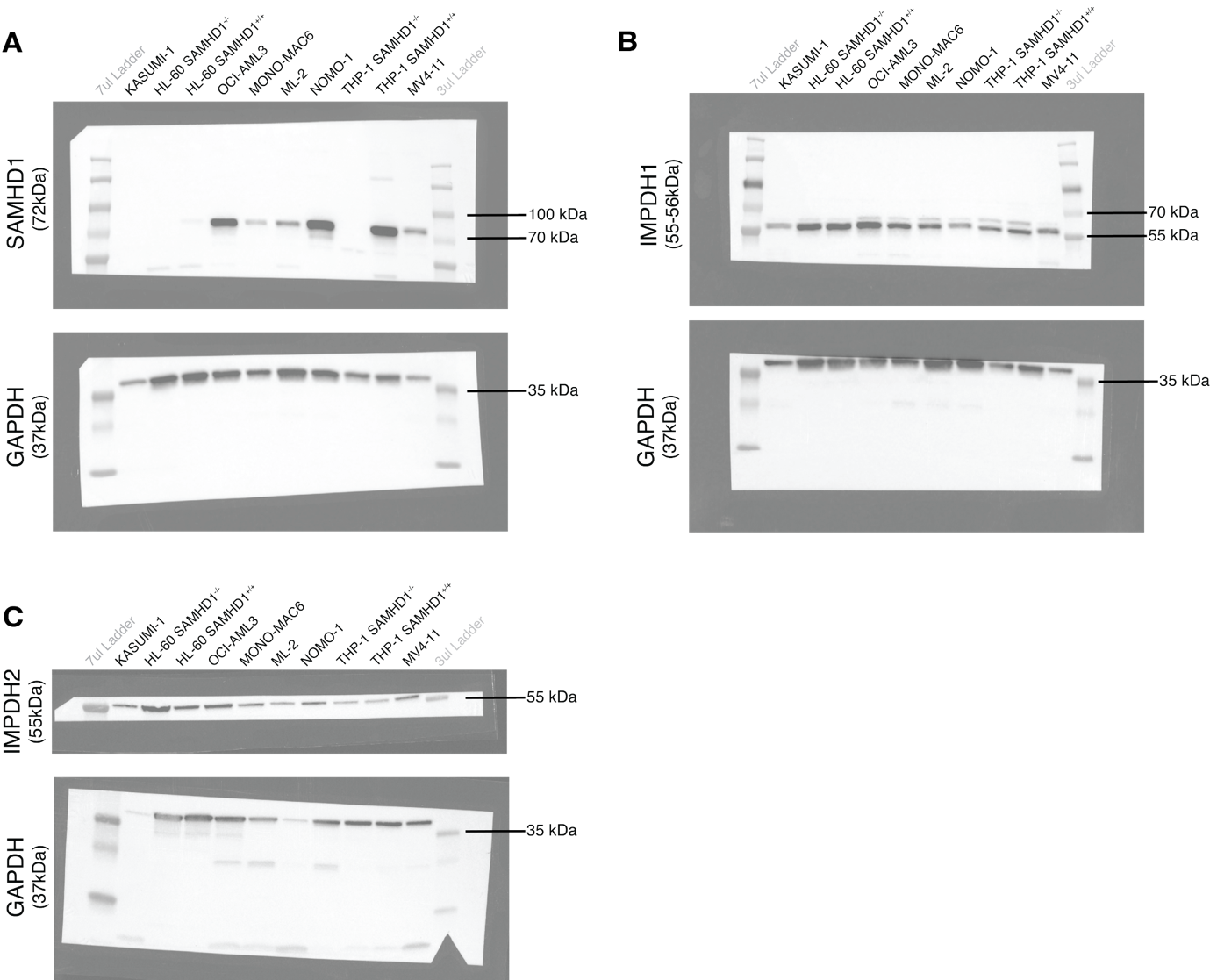
**

**Supplementary Figure 8:** **Expression of SAMHD1, IMPDH1, and IMPDH2 in AML cell lines.**

**(A-C)** Raw immunoblot images showing expression of SAMHD1 (A), IMPDH1 (B), and IMPDH2 (C) in the panel of AML cells. GAPDH was used as a loading control. These membranes correspond to the blots shown in Figure 1A.
